# Virulent strains of *Zymoseptoria tritici* suppress the host immune response and facilitate the success of avirulent strains in mixed infections

**DOI:** 10.1371/journal.ppat.1011767

**Published:** 2023-11-16

**Authors:** Alessio Bernasconi, Cécile Lorrain, Priska Flury, Julien Alassimone, Bruce A. McDonald, Andrea Sánchez-Vallet

**Affiliations:** 1 Plant Pathology, Institute of Integrative Biology, ETH Zürich, Zürich, Switzerland; 2 Centro de Biotecnología y Genómica de Plantas (CBGP/Universidad Politécnica de Madrid-Instituto Nacional de Investigación Agraria y Alimentaria/Centro Superior de Investigaciones Científicas (INIA/CSIC), Campus de Montegancedo, Pozuelo de Alarcón (Madrid) Spain; CAU: Christian-Albrechts-Universitat zu Kiel, GERMANY

## Abstract

Plants interact with a plethora of pathogenic microorganisms in nature. Pathogen-plant interaction experiments focus mainly on single-strain infections, typically ignoring the complexity of multi-strain infections even though mixed infections are common and critical for the infection outcome. The wheat pathogen *Zymoseptoria tritici* forms highly diverse fungal populations in which several pathogen strains often colonize the same leaf. Despite the importance of mixed infections, the mechanisms governing interactions between a mixture of pathogen strains within a plant host remain largely unexplored. Here we demonstrate that avirulent pathogen strains benefit from being in mixed infections with virulent strains. We show that virulent strains suppress the wheat immune response, allowing avirulent strains to colonize the apoplast and to reproduce. Our experiments indicate that virulent strains in mixed infections can suppress the plant immune system, probably facilitating the persistence of avirulent pathogen strains in fields planted with resistant host plants.

## Author summary

Mixed infections are commonly observed in crop fields and significantly influence the outcome of pathogen infections. Despite extensive research on plant resistance to pathogens, the impact of mixed infections within a plant host remains poorly understood. Traditionally, resistance studies have focused on single infections, neglecting the influence of co-existing strains and their behavior during infection. Our study investigates the interplay between virulent and avirulent strains of *Zymoseptoria tritici* and the capacity of this highly destructive wheat pathogen to manipulate the host immune system. We provide compelling evidence that virulent strains of *Z*. *tritici* suppress the plant immune response, thereby facilitating the early establishment and penetration of co-existing avirulent strains. Additionally, we demonstrate that the transcriptomic reprogramming of plants upon infection by avirulent strains is hindered by the presence of virulent strains. Overall, we demonstrated that virulent strains suppress the host immune response in mixed infections and that virulent strains promote the reproduction of avirulent strains in resistant host cultivars.

## Introduction

Complex biotic interactions within a plant can modulate the outcome of pathogen infections [1,2]. The dynamics of pathogen-host interactions involving mixed microbial infections can include cooperation, coexistence, and competition, where each infecting microbe may affect directly or indirectly the performance of the others [3]. Co-infecting microbes can compete directly through the secretion of molecules (e.g., antimicrobial metabolites) that affect the fitness of the competitors [1,4]. Within a shared host, microorganisms can also interact indirectly when the performance of each interacting microbe depends on the response of the host [5–7]. For instance, plant susceptibility or resistance triggered by a pathogen can affect the success of coinfecting microbes. In fact, infections by the fungi *Fusarium oxysporum* and *Zymoseptoria tritici* predispose wheat to be colonized by the bacteria *Pseudomonas fluorescens* and *Pseudomonas syringae*, respectively, through the suppression of host antimicrobial substances [8,9]. In particular, virulent strains of *Z*. *tritici* manipulate the immune response systemically and alter the wheat microbiome [8]. Distinctive plant immune responses are triggered upon infections produced by pathogens with different infection strategies and/or lifestyles, including biotrophs and necrotrophs [10]. Since plants are constantly challenged by an enormous diversity of pathogenic microorganisms with varying infection strategies [11–14], it is reasonable to expect that genotype-specific host immune responses may affect the infection outcome of co-infecting pathogen strains.

Comprehensive studies of plant immune responses to pathogen infections have mostly been conducted using experimental systems in which a single host genotype is infected by a single pathogen strain. Virulent pathogen strains colonize the host by secreting effectors that suppress host immunity [15–23]. To counteract pathogen colonization, plants evolved the ability to specifically recognize certain forms of effectors—or avirulence factors (Avrs)–through the production of resistance (R) proteins, and subsequently trigger an immune response that hinders the progression of the pathogen [24,25]. Pathogen strains harboring recognized Avrs are known as avirulent strains [26,27]. The host immune response can act locally, occurring only where the avirulent strain interacts with the host, but it can also spread from the infection site to produce a systemic response [28,29]. In mixed infections, virulent and avirulent pathogen strains may co-exist and affect the outcome of infection. In particular, the induction of host immunity caused by avirulent pathogen strains might suppress the development of co-infecting virulent strains. It is also possible that suppression of the host immune response by virulent pathogen strains might facilitate infection by co-infecting avirulent strains [14]. Our experiments aimed to better understand the balance between these processes.

*Z*. *tritici* is a genetically diverse fungal wheat pathogen in which several distinct strains typically coinfect the same leaf on a plant [14,30–32]. *Z*. *tritici* hyphae penetrate through the stomata and grow in the apoplast without producing symptoms for a period that lasts between 10 and 14 days under controlled conditions. Subsequently, it produces necrotic lesions on wheat leaves and forms both asexual (pycnidia) and sexual (pseudothecia) reproductive structures [33–35]. The septoria tritici blotch (STB) disease, caused by *Z*. *tritici*, leads to significant losses in wheat production worldwide and is managed mainly using host resistance and fungicides. Genetic control of STB is based on wheat resistance genes, of which 22 have been identified so far [36–39] and two (Stb6 and Stb16) have been cloned and functionally characterized [37–40]. Stb6 recognizes avirulent isoforms of the fungal effector AvrStb6, following the gene-for-gene interaction model [41,42], leading to the induction of an immune response that prevents the progression and asexual reproduction of AvrStb6-expressing strains [40–42]. The mechanisms by which resistant cultivars hinder progression of avirulent strains remain mostly unknown, though recent work demonstrated that stomatal immunity and production of reactive oxygen species (ROS) are likely to play key roles in preventing infection of avirulent strains harboring AvrStb6 and AvrStb16 [43,44]. Field populations of *Z*. *tritici* carry a diverse array of AvrStb6 isoforms, with virulent and avirulent strains typically coexisting in the same field [45]. Remarkably, it has been demonstrated that avirulent strains are capable of reproducing sexually with co-infecting virulent strains despite not being able to infect a resistant host [41,46]. It has yet to be determined if and how mixed infections affect asexual reproduction.

In this work, we demonstrate that an avirulent *Z*. *tritici* strain can penetrate, colonize and reproduce asexually on a resistant host in a mixed infection with virulent strains. Microscopic observations of the disease progress indicate that virulent *Z*. *tritici* strains facilitate the infection and asexual reproduction of co-infecting avirulent strains. We further demonstrated, using a comparative transcriptomic approach, that virulent strains suppress avirulent strain-triggered host immune responses. Our findings indicate that mixed infections promote the asexual reproduction of avirulent pathogen strains in resistant plant populations.

## Results

### Mixed infections facilitate the asexual reproduction of avirulent strains

**Fig 1:**
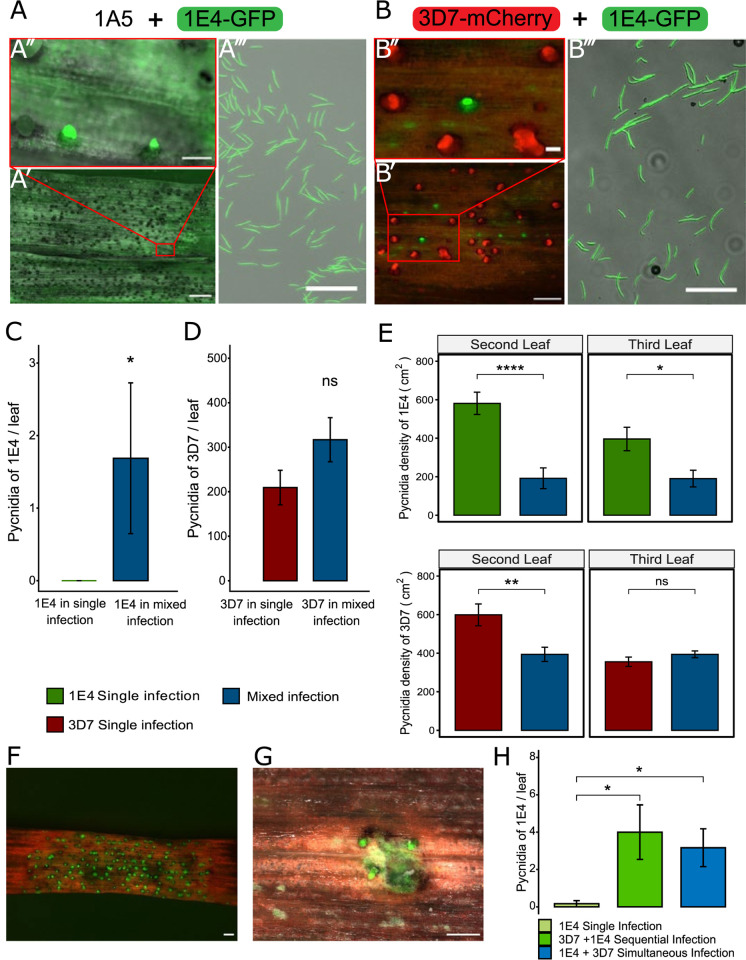
*Zymoseptoria tritici* avirulent strain 1E4 can reproduce in mixed infections with virulent strains. **A)** The avirulent strain 1E4-eGFP produces pycnidia on the resistant wheat cultivar Chinese Spring 19 days post infection (dpi) in mixed infections with the virulent strain 1A5. A′ and A″: Leaf surface with mature fluorescent pycnidia and extruded cirri of 1E4-eGFP. Scale bar: 1 mm (A′) and 100 μm (A″). **B)** The avirulent strain 1E4-eGFP produces pycnidia in the presence of the virulent strain 3D7-mCherry on the resistant cultivar Chinese Spring at 20 dpi. B′ and B″: Pycnidia distribution patterns of 3D7-mCherry and 1E4-eGFP. Scale bar: 500 μm (B′) and 100 μm (B″). The red boxes in A′ and B′ indicate the zoom out area displayed in A″ and B″. A‴ and B‴: pycnidiospores collected from the eGFP-labeled pycnidia are from 1E4-eGFP. Scale bar: 100 μm **C)** 1E4 produces more pycnidia at 20 dpi in mixed infections with the virulent strain 3D7 than in single infections. Barplots show the average number of 1E4 pycnidia per leaf formed. Error bars represent the standard error of the mean. Biological replicates are shown in S1 Fig. Significant differences between single and mixed infections according to one-tailed Kruskal-Wallis test are indicated (* p < 0.05). **D)** The virulent strain 3D7 produces the same number of pycnidia per leaf regardless of the presence of the avirulent strain 1E4. Barplots show the average number of 3D7 pycnidia per leaf. Error bars represent the standard error of the mean. Biological replicates are shown in S1 Fig. There are no significant differences between single and mixed infections according to ANOVA followed by the Tukey’s HSD post-hoc test (p < 0.05). In C) and D) a total of 16 third leaves per treatment were analyzed. **E)** Barplots of the average number of pycnidia per cm^2^ of leaf from 1E4-eGFP (top panel) and 3D7-mCherry (bottom panel) produced on detached leaves of Chinese Spring in single and mixed infections. Error bars represent the standard error of the mean of three biological replicates. Asterisks indicate significant differences according to student test (* p < 0.05 and ns = not significant). **F, G)** Pictures of pycnidia of 1E4-eGFP at 20 dpi in Chinese Spring leaves damaged with celite **F)** or poked with a needle **G)** prior to inoculation. Scale bars indicate 500 μm. The experiment was performed twice. **H)** Barplots of the average pycnidia density per leaf of 1E4 infected alone, infected 7 days after inoculation with 3D7 (asynchronous) and infected simultaneously with 3D7 in the cultivar Chinese Spring. Infections were evaluated 20 days after inoculation of 1E4. Data is from three biological replicates. Asterisks indicate significant differences according to Anova followed by Tukey’s HSD test (p < 0.05).

We first investigated if avirulent strains can asexually reproduce in the presence of virulent strains. We simultaneously co-infected the Stb6-containing resistant cultivar Chinese Spring with two strains harboring either virulent (3D7 or 1A5) or avirulent (1E4) isoforms of AvrStb6. To discriminate between strains in mixed infections, we used eGFP-labeled 1E4 and mCherry-labeled 3D7. These fluorescent lines were previously obtained and tested [14]. We additionally used an unlabelled 1A5 strain. We observed 1E4-eGFP cirri on leaves co-infected with 1A5 or 3D7-mCherry (Fig 1A and 1B). Pycnidia from the avirulent strain 1E4-eGFP were rarely observed in single infections (3 pycnidia observed in 48 leaves), while the reproductive success of 1E4-eGFP was higher (according to Kruskal-Wallis test; with 67 pycnidia observed in 48 leaves) in mixed infections with virulent strains (Figs 1C, 1H; S1 and Table A in S1 Text). The number of pycnidia produced by the virulent 3D7-mCherry strain was the same in both single and mixed infections (Figs 1D; S1 and Table A in S1 Text). We conclude that mixed infections of avirulent and virulent strains do not affect the spore production of the virulent strain, but significantly increase the reproductive success of the avirulent strain.

We hypothesized that the increase in the reproductive rate of the avirulent strain 1E4 in co-infections could be due to direct interactions between the strains, the host environment, plant cell damage produced by the virulent strain or to the suppression of the immune response by the virulent strain. We first investigated how the host environment influenced the interaction between virulent and avirulent strains by performing infection assays on detached leaves [47]. Unlike on intact leaves, 1E4-eGFP produced pycnidia on detached leaves, suggesting that detached leaves do not express the full range of defenses or suffer early senescence which facilitates the infection by the avirulent strain (Fig 1E). The avirulent strain 1E4-eGFP achieved significantly higher pycnidia density in single infections than in mixed infections on both second (p < 0.001) and third detached leaves (p = 0.015; Fig 1E and Table B in S1 Text). Pycnidia density of the virulent strain 3D7-mCherry was reduced on the second leaf (p = 0.007), but not on the third leaf (p = 0.215) by the presence of 1E4-eGFP (Fig 1E, below and Table B in S1 Text). These results indicate that asexual reproduction on detached leaves is potentially limited by competition [14] between avirulent and virulent strains. We consider that on detached leaves, the immune response is suppressed since the number of pycnidia produced by both strains was very high. In contrast, in intact leaves we observed that asexual reproduction of the avirulent strain is favored by the presence of the virulent strain (Fig 1C). We therefore suggest that interactions between strains are conditional on the host environment since they most probably depend on host senescence and host immunity.

We next investigated whether plant cell damage produced by the virulent strain benefited the avirulent strain by determining whether mechanical leaf damage affects the reproductive success (i.e., pycnidia production) of the avirulent strain. Chinese Spring leaves were damaged with celite or poked with a needle before infection with 1E4-eGFP. We observed that the avirulent strain produced pycnidia on the leaf regions damaged with celite (approximately 400 pycnidia/leaf; S2 Fig) and on 20% of the areas poked with a needle. However, no pycnidia of the avirulent strain were formed on the intact regions of the leaf (Figs 1F and 1G; S2 and Table C in S1 Text). The results suggest that damage to the leaf epidermis allows the fungus to penetrate the plant tissues and produce pycnidia. We further evaluated whether if, in addition to leaf damage and necrosis induced during infection, host immune suppression by the virulent strain also promotes the colonization by the avirulent strain by infecting Chinese Spring plants first with the virulent strain 3D7-mCherry and re-infecting them seven days later with the avirulent strain 1E4-eGFP. In this experiment, 1E4-eGFP infection was initiated during the 3D7-mCherry latent phase and only 5 days before the beginning of the 3D7-mCherry necrotrophic phase. We expected that if additional factors, other than necrosis, facilitate 1E4 infection, we would also see an advantage for 1E4 reproduction in asynchronous infections. As controls, we conducted single infections with 1E4-eGFP and made simultaneous infections, in which both strains were co-inoculated as previously indicated (Fig 1A–1C). In asynchronous co-infections, 1E4-eGFP produced more pycnidia in mixed infections than in single infections (p = 0.023; Fig 1H). The prior infection by the virulent strain did not significantly increase the number of pycnidia of the avirulent strain, compared to the simultaneous infection (Fig 1H and Table D in S1 Text). This result suggests that the capacity of 1E4-eGFP to reproduce is affected by other factors besides the time in which the necrotrophic phase of 3D7-mCherry is initiated. We postulated therefore, that other mechanisms beyond the necrosis induced by the virulent strain, such as suppression of plant resistance by the virulent strain, might be involved.

### The virulent strain facilitates apoplast colonization of the avirulent strain

**Fig 2:**
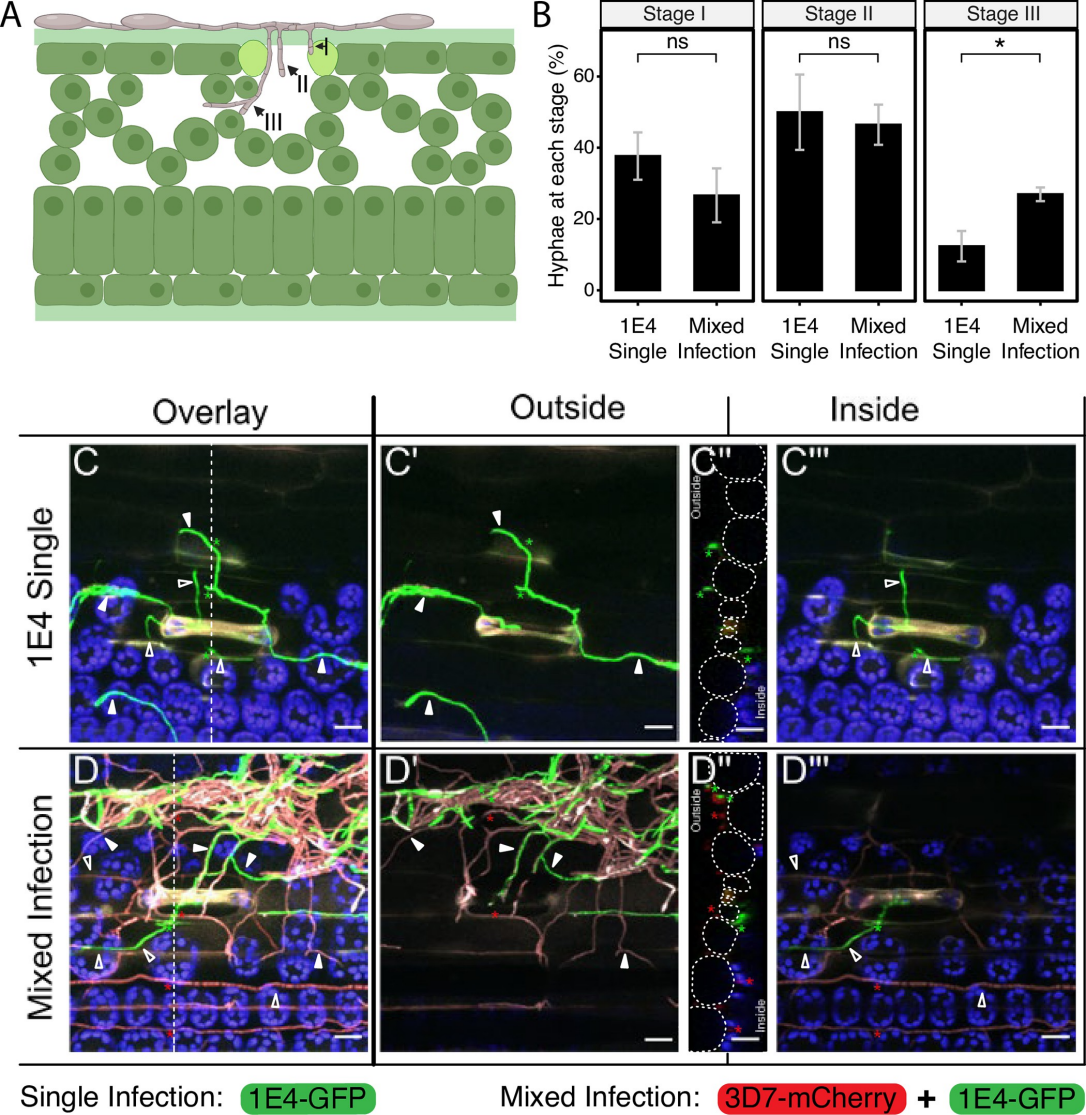
*Zymoseptoria tritici* avirulent strain reaches the mesophyll more frequently in mixed infections with a virulent strain. **A)** Hyphae progression of the avirulent strain (1E4 labeled with eGFP) in the resistant wheat cultivar Chinese Spring was estimated by defining three infection stages: I, the hyphae attempt to penetrate the stomata, but it does not reach the substomatal cavity; II, the hyphae reach the substomatal cavity; III, the hyphae reach the mesophyll cells. Created with Biorender. **B)** Hyphae from the avirulent strain 1E4 reach the mesophyll cells more frequently in mixed infections with the virulent strain 3D7 (labeled with mCherry) than in single infections. The percentage of hyphae at each infection stage was estimated at 11 days after infection of Chinese Spring plants with 1E4-eGFP or with a mixture of 1E4-eGFP and 3D7-mCherry. Bars represent average of three biological replicates, and error bars show the standard error of the mean. In total between 10 and 29 observations per treatment and replicate were made, with a total of 64 observations made for mixed infections and 65 for 1E4 single infections. Asterisks indicate statistical differences according to two-tailed student’s test (P < 0.05). An independent repetition of this experiment is shown in S3 Fig **C, D)** Maximum projection overlays of Z stack acquisitions illustrating stage III penetration events of 1E4 (1E4-eGFP, green) in single infections or in mixed infections with the virulent strain 3D7 (3D7-mCherry, red) (C and D panels respectively). Dotted lines across C and D mark the position of the orthogonal views (yz) displayed in C″ and D″. Dotted circles on the orthogonal view (C″, D″) delimit epidermal cells outlines. C′ and D′ panels display the outside section from the Z stack acquisition. C‴ and D‴ panels display exclusively the plant inner tissues from the Z stack acquisition. Asterisks pinpoint hyphae visible on the orthogonal views. Full and empty arrowheads indicate outer and inner hyphae respectively. Chloroplast autofluorescence is displayed in blue in all C and D panels. The scale bars represent 20 μm.

To test whether 1E4 is favored in mixed infections with virulent strains at the early stages of the infection, before symptoms become visible, we monitored hyphal plant penetration using confocal microscopy at 11 dpi. As previously reported for *Z*. *tritici* avirulent strains [43,44,48], we observed that not all 1E4 hyphae penetrated the stomata, supporting the theory that AvrStb6-triggered resistance occurs at the penetration step. Of the total number of hyphae that were in contact with stomata, we estimated that 41% and 39.7% (in each of the replicates) of 1E4 hyphae grew over the stomata or did not penetrate in single and mixed infections, respectively. We divided the progression of avirulent strains into 3 phases: stage I occurs when hyphae attempt to penetrate through the stomata, but do not reach the substomatal cavity (unsuccessful attempt); stage II occurs when hyphae reach the substomatal cavity; and stage III occurs when hyphae enter into the substomatal cavity and reach the apoplastic space in the mesophyll (Fig 2A). At the first stages of penetration (I and II), there were no differences in the performance of the avirulent strain in single and mixed infections with the virulent strain (Figs 2A and 2B; S3 and Tables E and F in S1 Text). However, the number of hyphae from the avirulent strain 1E4-eGFP that grew in the apoplastic space (stage III) was significantly higher (p = 0.029) in mixed infections compared to single infections (Figs 2B–2D, S3 and Tables E and F in S1 Text). These results indicate that host colonization by the avirulent strain at the early stages of infection is promoted by co-infections with the virulent strain. Our data suggest that co-infection enables colonization of the apoplastic space during the asymptomatic phase of the infection, leading to an increase in asexual reproduction of the avirulent strain.

### Virulent strains of *Z*. *tritici* manipulate the plant immune response in mixed infections

**Fig 3:**
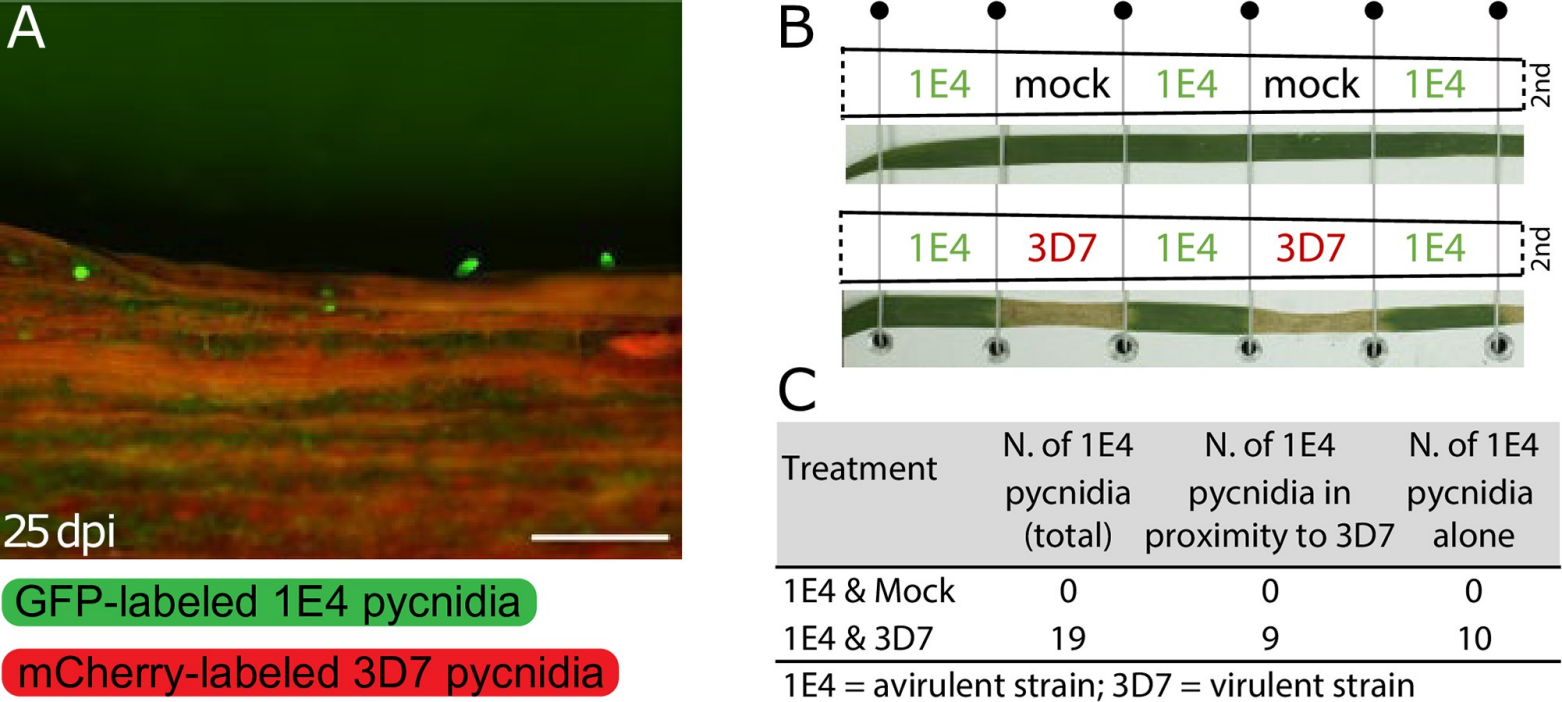
Local and distant infections favor the reproduction of the avirulent strain of *Zymoseptoria tritici*. **A)** The avirulent and the virulent strains were inoculated onto distant regions of wheat leaves. An example of pycnidia produced by 1E4 labeled with eGFP in positions distant from the virulent strain 3D7 tagged with mCherry (not in the picture). Scale bars: 500 μm **B)** Scheme of the distant infection (Side-by-side infection) performed on second leaves placed horizontally on a metal plate and kept in this position with elastic threads. Elastic threads segmented the leaf into 7 2-cm length sections. Below each scheme, a representative picture of leaves infected side-by-side with 3D7-mCherry and 1E4-eGFP or treated with the mock-solution and 1E4-eGFP is shown. Segments infected with 3D7-mCherry show necrotic lesions between the two elastic threads. **C)** Table summarizing the results of the side-by-side experiment indicating the number of pycnidia of 1E4-eGFP in distant infections with mock or with 3D7-mCherry. The number of pycnidia from 1E4-eGFP found in the same picture as 3D7-mCherry pycnidia (N. of 1E4 pycnidia in proximity to 3D7), or completely isolated from pycnidia of 3D7-mCherry (N. of 1E4 pycnidia alone) is indicated.

To determine if the beneficial effect of the virulent strain on the avirulent strain also acts in distant regions of the leaf, we performed an infection assay in which the virulent and avirulent strains were physically separated (Fig 3). In total, 19 pycnidia of 1E4-eGFP were counted in the leaf regions adjacent to 3D7-mCherry inoculated areas, while no 1E4-eGFP pycnidia were found on leaf sections adjacent to mock-treated areas (Fig 3A and 3C). Out of the 19 pycnidia identified in regions infected with 1E4-eGFP, 10 were completely separated from the leaf region colonized by the virulent strain and 9 were located in close proximity to pycnidia of the 3D7-mCherry strain (Fig 3C). Although we cannot discard the possibility that 3D7 hyphae spread to the 1E4-infected area, these findings indicate that infection by virulent strains of *Z*. *tritici* increases plant susceptibility, and this immune suppression is not restricted to the infection site, enabling infection by avirulent strains at separate locations.

**Fig 4:**
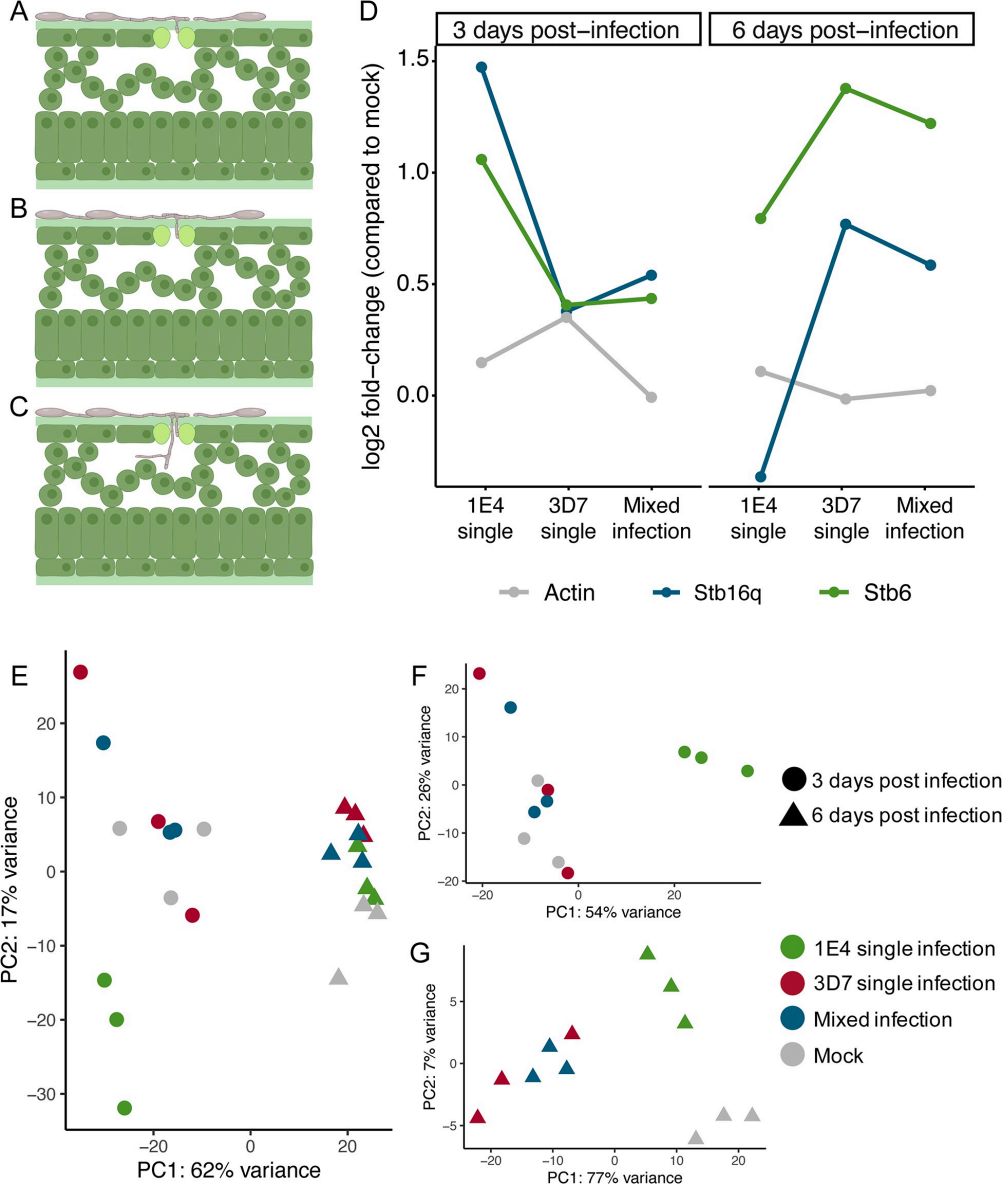
Mixed infections and the virulent strain trigger a similar host transcriptome response that differs from that of the avirulent strain. Schematic representation of the infection stages of *Zymoseptoria tritici*: **A)** At 3 days post infection (dpi) 1E4 and 3D7 grow mainly epiphytically with few successful examples of stomatal penetration. **B)** At 6 dpi, the avirulent strain 1E4 in single infections remains mostly growing on the leaf surface and the penetration attempts are blocked by the host immune response. **C)** At 6 dpi hyphae of the virulent strain 3D7 in single infection have penetrated the stomata and started to grow in the sub-stomatal cavity. A, B and C were created with Biorender. **D)** Expression (log2 fold-change compared to mock) of the major resistance genes *Stb6* and *Stb16q* (Tables E and F in S1 Text) compared to the *Actin* housekeeping gene corresponding to the three infection treatments (1E4 single, 3D7 single and mixed infection) at 3 and 6 dpi. **E)** Principal component analysis (PCA) of DESeq2 normalized read counts at 3 (triangles) and 6 dpi (circles) for the four treatments, including: uninfected leaves (grey), leaves infected by the avirulent strain 1E4 (green), leaves infected by the virulent strain 3D7 (red), and leaves coinfected with both 1E4 and 3D7 (blue) strains. Read counts were normalized using the size factor method and rlog transformed before performing the PCA. The PC1 (x-axis) explains 62% of the variance between treatments and time-points, while the PC2 (y-axis) explains 17% of the variance. **F)** PCA including only samples harvested at 3 dpi. Samples are placed along two PC axes explaining 54% (x-axis) and 26% (y-axis) of the variance. **G)** PCA including only samples harvested at 6 dpi. Samples are placed along two PC axes explaining 77% (x-axis) and 7% (y-axis) of the variance.

To better understand the host immune response upon recognition of an avirulent strain in single and in mixed infections, we compared the transcriptomic profiles of wheat leaves infected with the virulent strain, the avirulent strain or a mixture of both strains at 3 and 6 dpi. At 3 dpi the hyphae of both strains are mostly growing on the leaf surface and attempting to penetrate through the stomata [19,37,48]. We considered therefore, that at early time points, the virulent and the avirulent strain are in the same infection stage (Fig 4A and 4B). Additionally, since 1E4-eGFP alone can barely penetrate further than stage III (Fig 2B), we hypothesized that it should trigger plant immunity at early infection stages. We included in our transcriptome analysis a later time point (6 dpi) to evaluate if the effect of the infection was maintained over time. Based on previous works [37,48], at 6 dpi some virulent strain hyphae have already penetrated the substomatal cavity and initiated the colonization of the apoplast (Fig 4C). At both time points, we obtained a lower number of mapped reads for 1E4 (61,435 at 3 dpi and 80,021 at 6 dpi) than for 3D7 (97,779 at 3 dpi and 159,585 at 6 dpi), which might indicate that the host immune response hindered the progression of the avirulent strain, although we cannot discard the possibility that 1E4 has a lower epiphytic growth (S4 Fig). We monitored the expression of two previously identified resistance genes in *Z*. *tritici*, *Stb6* and *Stb16q* [37,40]. At 3 dpi, both genes were highly expressed during the infection by the avirulent strain, but not when 3D7 was present in single or in mixed infections (Fig 4D). In contrast, we observed the opposite effect at 6 dpi. At this time point, both resistance genes were more highly expressed in plants infected with 3D7 compared to the response upon 1E4 treatment. These results suggest that the defense-related genes induced at early stages of the infection in response to the avirulent strain are not up-regulated when the virulent strain is present.

To obtain a broader overview of the differences in the transcriptomic profiles for the three treatments, we performed a principal component analysis (PCA) of the normalized read counts. We observed a clear distinction in the wheat transcriptomic profiles between 3 and 6 dpi (62% of variance explained on the PC1 axis; Fig 4E). We considered that these differences are partially due to the developmental stage of the plant since we also observed a distinct response in mock-inoculated plants at both time points (Fig 4E). Plants infected by only the avirulent strain displayed a distinctive transcriptomic profile compared to the other infection treatments and water-sprayed leaves (mock treatment) at both 3 and 6 dpi (Fig 4F and 4G). At these time points, the transcriptomic profile of leaves infected with the virulent strain and in mixed infections were not distinct and clustered together in the PCA (Fig 4F and 4G). The similarity of the transcriptomic profiles of plants infected with the virulent strain in single and in mixed infections (Fig 4F and 4G) indicates that the transcriptional response of the host was independent of the presence of the avirulent strain in mixed infections. These results demonstrate that 1E4 induces a transcriptomic reprogramming and suggest that 3D7 overshadows the response of the avirulent strain in mixed infections.

**Fig 5:**
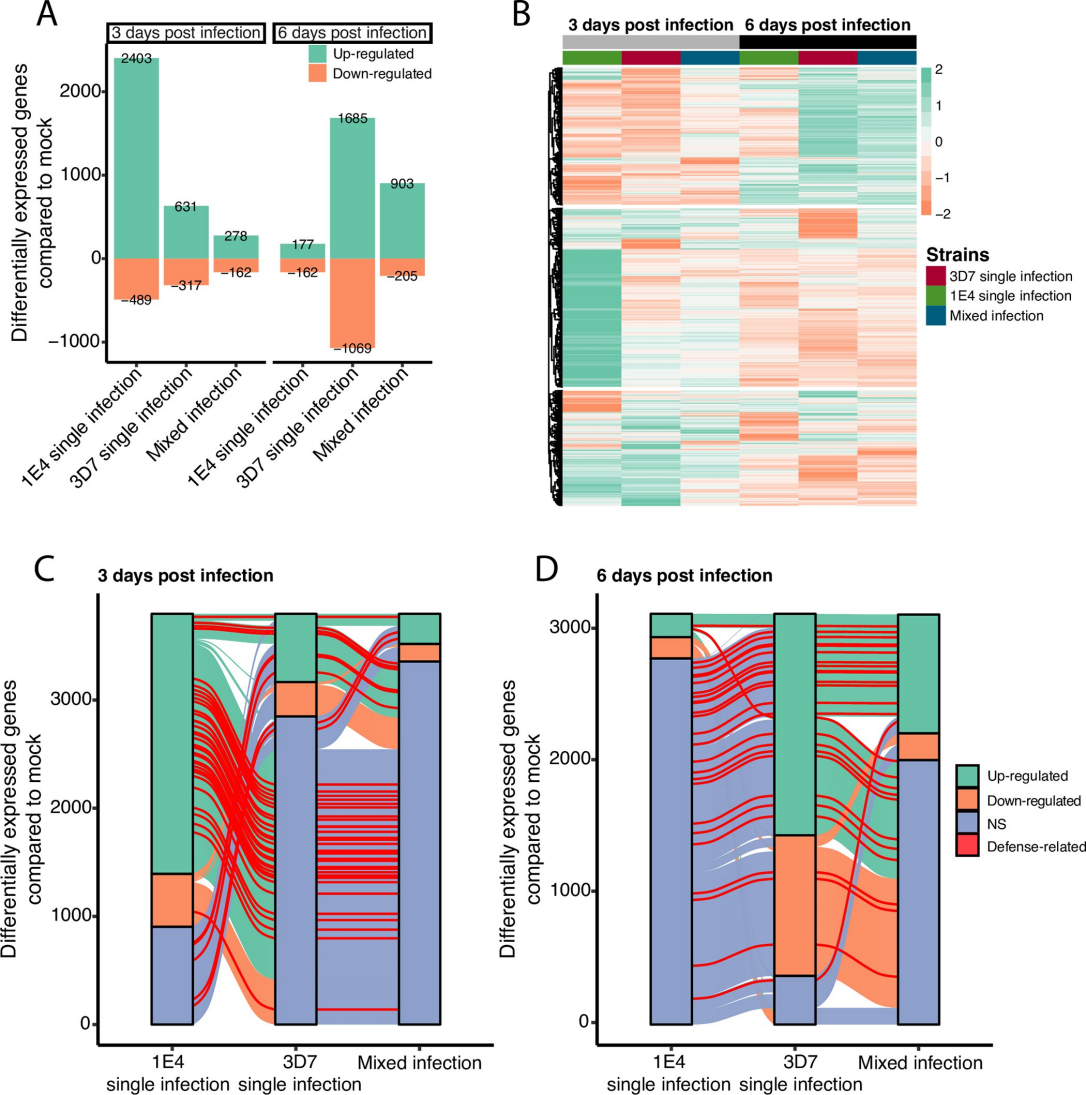
Transcriptome analysis of wheat indicates that the virulent strain of * Zymoseptoria tritici* controls avirulence factor-triggered plant resistance. **A)** Number of differentially expressed genes (DEGs) in wheat upon each treatment compared to uninfected leaves identified with DESeq2 (p-adjusted < 0.05). Green bars represent up-regulated DEGs (log2 fold-change > 0) and orange bars represent down-regulated genes (log2 fold-change < 0). **B)** Transcript profiling across the three treatments and two-time points of the 6256 genes differentially expressed in at least one condition compared to uninfected plants. Transcript expression is represented in row scaled log2-fold change using uninfected leaves as a reference, from up-regulated genes (green) to down-regulated genes (orange). DEGs are grouped by hierarchical clustering based on log2-fold change values. **C, D**) Alluvial plot of differentially expressed genes: up-regulated in green, down-regulated in orange and non-significant DEGs in blue; at **C)** 3 dpi and **D)** 6 dpi and across each treatment. Genes with "defense-related" GO predictions are highlighted in red. The “defense-related” protein sequences were manually checked for similarity in Arabidopsis using BLASTP.

To further explore the plant responses in single and mixed infections, we analyzed the differentially expressed genes (DEGs) in infected wheat leaves with the virulent strain, the avirulent strain or both (mixed-infection) compared to mock-treated plants at each time point. We counted 2892 DEGs (log2-fold change > 0 and p-value adjusted < 0.05) during the infection of the avirulent strain compared to the mock-infected plants at 3 dpi (Fig 5A). In contrast, the leaves infected by the virulent strain, or the mixed infection showed fewer DEGs compared to the leaves infected by the avirulent strain, with 948 and 440 DEGs, respectively. At 6 dpi, the numbers of DEGs increased in both the virulent strain infection and the mixed infection with 2754 (with 61% of genes up-regulated) and 1108 (with 81% of genes up-regulated), respectively (Fig 5A), while 339 DEGs were identified upon infection with the avirulent strain. Taken together, these results show distinctive transcriptional responses between timepoints and infection treatments.

Hierarchical clustering of the expression profiles of all DEGs revealed that the majority of genes up-regulated in the 1E4 single infection at 3 dpi are not differentially expressed in the 3D7 and mixed infections (Figs 5B and S5). We next explored in detail which DEGs were shared among the different treatments at each timepoint. The set of up-regulated genes at 3 dpi during infection by the avirulent strain 1E4 was different from the set of genes up-regulated in the virulent or mixed infections (Figs 5C and S5). Among the 2403 up-regulated DEGs in response to the avirulent strain at 3 dpi, only 9.5% and 4% were also up-regulated in leaves exposed to virulent and both strains, respectively (Figs 5C; S5, Tables G, H, I, J and K in S1 Text). Similarly, at 6 dpi we observed a common transcriptomic pattern in plants infected with the virulent strain either in single or mixed infections. In particular, among the 1685 up-regulated DEGs in response to a 3D7 single infection at 6 dpi, 46% were also up-regulated in the mixed infection, while only 7% were up-regulated in the infection by 1E4 (Figs 5D and S5). These results show that a similar plant response occurs when leaves are exposed to a virulent strain in a single or mixed infection. Since the avirulent strain leads to a distinct transcriptomic profile, we conclude that the virulent strain masks the presence of the avirulent strain in a mixed infection.

Leaves infected with the avirulent strain 1E4 included 48 up-regulated genes at 3 dpi that have Gene Ontology annotations related to “defense response” (S6 Fig; Tables H, I, K, L, N in S1 Text). Only 8 and 4 genes of the same category were up-regulated in leaves infected with the virulent strain in single or mixed infections, respectively (Figs 5C and S6; Tables I, J, K in S1 Text). Therefore, the defense-related genes induced by a 1E4 single infection were mostly not up-regulated in a 3D7 single infection or a mixed infection (Fig 5C and Table K in S1 Text). We also observed enrichment of GO categories representing downstream signaling in plant immune responses such as "response to abscisic acid", "response to jasmonic acid" and "response to salicylic acid" (Tables L, M in S1 Text) in leaves infected by the avirulent strain. At 6 dpi, 34 and 22 defense response-related genes were up-regulated in plants infected with the virulent strain 3D7 or both strains, respectively, while only 2 were up-regulated in plants infected with only the avirulent strain 1E4 (Figs 5D and S6; Tables H, I, J, K, M, N in S1 Text). Overall, the results obtained with the transcriptomic analysis indicate that the avirulent strain triggered an early resistance response in single infections, but not in mixed infections. This indicates that virulent *Z*. *tritici* strains are able to suppress the immune response while colonizing the host.

## Discussion

The colonization and reproductive success of avirulent strains on resistant cultivars are severely limited in single infections in which only one strain attempts to colonize the host [24,34,49]. In this work we demonstrated that mixed infections provide an advantage to the growth and reproduction of avirulent strains on resistant hosts. We found that this advantageous effect begins at early stages of the infection process. Our findings provide new insights into the factors that could contribute to the maintenance of diversity in pathogen populations and highlight how the complex nature of pathogen-plant interactions can challenge the development of sustainable control of wheat diseases [50]. In mixed infections, virulent strains of *Z*. *tritici* can suppress the plant immune system and allow colonization and reproduction by otherwise avirulent pathogen strains, enabling the persistence of avirulent strains in fields planted to resistant cultivars.

*Z*. *tritici* is an apoplastic pathogen. Upon germination of sexual and asexual spores, fungal hyphae grow on the leaf surface and penetrate through stomata. Recognition of avirulence factors hampers the reproduction of this pathogen [41,42,48]. Furthermore, it was recently demonstrated that the penetration rate of avirulent strains harboring either AvrStb16 or Avr3D1 is strongly impaired in resistant hosts and that stomata are more closed upon AvrStb6 and AvrStb16 recognition [37,43,44,48]. In this work, we also observed that early stages of the infection of 1E4 (harboring AvrStb6) are hampered, with only some hyphae reaching the apoplastic space outside of the substomatal cavity. These results support the hypothesis that AvrStb6 recognition occurs during penetration. We additionally showed that virulent strains facilitate penetration of 1E4, suggesting that 3D7 suppresses the immune response early during infection. However, we cannot discard the possibility that the interaction between *Z*. *tritici* and the host already occurs during *Z*. *tritici* epiphytic growth. The epiphytic growth of *Z*. *tritici* has been shown to be critical for its life cycle [46,51,52]. In fact, avirulent isolates of *Z*. *tritici* have been shown to proliferate epiphytically on resistant plants and induce early defense responses [53]. The proliferation of AvrStb6-expressing strains on the leaf surface together with the frequently occurring genetically diverse STB infections, might facilitate on the one hand sexual reproduction, as previously shown [41,46] and on the other hand penetration through the stomata and pycnidia formation, as shown in this work. Additionally, we showed that 1E4 pycnidia in detached leaves are formed but in contrast to what we found in intact plants, the presence of 3D7 hindered asexual reproduction of 1E4. Pycnidia production of 3D7 was reduced by the presence of 1E4 on detached second leaves, but not on detached third leaves. We suggest that the plant immune response and/or hormonal balance might be altered on detached leaves, enabling pycnidia formation of the avirulent strain and influencing the competitive capacities of different *Z*. *tritici* strains. We observed that wounding also facilitates asexual reproduction of avirulent strains similar to what was shown earlier for AvrStb16-harboring strains [43]. Overall, these results highlight that even when avirulent strains are detected and their infection is prevented, they can still survive on the leaf surface and their co-existence with virulent strains or leaf damage can facilitate the persistence of avirulent strains in *Z*. *tritici* populations [46,53]. Although the avirulent strain formed many fewer pycnidia than the virulent strain, this ability of avirulent strains to reproduce in mixed infections will facilitate the survival of avirulent strains and enable the persistence of avirulent alleles in fields planted to resistant hosts. A recent analysis indicated that a moderate infection of STB (44% of leaves infected) will produce approximately one billion pycnidia per hectare [32]. If mixed infections by virulent strains on resistant hosts enable 1% of the pycnidia to be contributed by avirulent strains, this would yield 10 million avirulent pycnidia per hectare. While this would represent only a small contribution to the overall epidemic, it would provide ample opportunity for avirulent strains to contribute to the evolutionary potential of the pathogen population through both sexual and asexual reproduction.

The transcriptomic analyses revealed that early host defense responses activated by the avirulent strain are not induced in co-infections with a virulent strain. In gene-for-gene interactions, early detection of a pathogen has been described as the key factor affecting the efficient suppression of plant pathogens [54]. In accordance, the avirulent strain 1E4 infection progress was arrested at the early stages of infection, during penetration of the host. We additionally found that the avirulent strain induced a higher number of defense-related genes at the early stages of the infection compared to virulent strains. For example, *Stb6* was slightly but significantly up-regulated at 3 dpi in the incompatible interaction, but was not differentially expressed in infections with the virulent strain. We expected that the avirulent strain 1E4 would be detected because of the presence of the Stb6 resistance protein in Chinese Spring [40–42], regardless of the presence of the virulent strain. Instead, only 4% of the genes that were up-regulated in the incompatible interaction at 3 dpi were also differentially expressed in the mixed infection. We hypothesize that virulent strains of *Z*. *tritici* suppress the immune response in mixed infections, either by preventing the recognition of avirulent strains or by suppressing the resistance response triggered by avirulent strains attempting to penetrate. We suggest that this suppression facilitates infection by 3D7, but also enables infection by otherwise avirulent strains of the same species. In accordance, Suffert and collaborators [55] suggested that fast colonizer virulent strains suppress the immune response, facilitating the infection of other slower colonizing virulent isolates and, subsequently, favoring sexual reproduction. This immune suppression should occur at early stages of the infection, since we observed an enhanced penetration rate for the avirulent strain, and it has an effect in distant regions of the leaf, since we observed isolated 1E4 pycnidia. This remarkable finding is in accordance with previous reports that *Z*. *tritici* enables the colonization of non-pathogenic species and other components of the microbiome of wheat [8,56]. In earlier work, Seybold and collaborators [8] demonstrated that virulent strains of *Z*. *tritici* enabled infection by non-adapted *Pseudomonas* sp. through the suppression of the host immune response. Remarkably, they demonstrated that this suppression of the immune response was not only local but also systemic, since the effect was maintained in distant leaves. They also demonstrated that *Z*. *tritici* affected leaf microbiota [8]. Our experiments add further support to the hypothesis that successful pathogens manipulate the host immune system and influence secondary infections by avirulent strains. In accordance with previous publications [8,56], we suggest that successful pathogens might enable colonization by other microbes, including beneficial microbes, non-pathogenic and pathogenic species, and avirulent strains of pathogens [3,8]. We postulate that virulent *Z*. *tritici* strains secrete an arsenal of effectors which manipulate the host immune response, rendering the plant more susceptible to colonization by coexisting pathogens and other microorganisms. We believe that, similar to other pathogens [57], host immune suppression is a general mechanism used by *Z*. *tritici* to infect wheat plants.

Mathematical models suggest that avirulence alleles are likely to disappear in natural populations when resistant hosts dominate due to the evolutionary pressure exerted by resistance genes [58–60]. Models also indicate that mixed infections are likely to impact the evolution of virulence [7]. Previous work demonstrated that avirulent and virulent strains of *Z*. *tritici* are capable of sexually recombining on resistant cultivars, providing a mechanism to maintain avirulent *AvrStb6* alleles in *Z*. *tritici* populations [41]. Our experiments showed that mixed infections of *Z*. *tritici* can facilitate the asexual reproduction of avirulent strains. These results provide another possible mechanism to maintain avirulent effector alleles at low frequencies in resistant host populations. We speculate that the same mechanisms will enable avirulence alleles to be maintained in resistant host populations for many plant pathogens.

## Materials and methods

### Plant and fungal material

All experiments were conducted with the wheat cultivar Chinese Spring (*Triticum aestivum* L., Delley Semences et Plants SA, DSP, Delley, Switzerland), which contains the resistance gene *Stb6*. Depending on the experiment, 8 or 16 seedlings were grown in square 11 x 11 x 12 cm plastic pots (Bachmann Plantec AG, Switzerland) containing peat soil (Jiffy soil substrate GO PP7, Netherlands) for 17 days prior to infection. 10-day-old plants were fertilized with 2 litres of fertilizer solution per 15 pots (2 mL L^−1^, Wuxal Universal-Dünger, Maag-Garden, Switzerland). Growing conditions were the following: 16 h of light, 70% relative humidity, temperature of 18°C during the day and 15°C during the night, and light intensity of 12 kLux.

The ST99CH_3D7 and ST99CH_1A5 (abbreviated as 3D7 and 1A5, respectively) strains of *Z*. *tritici* are virulent on cultivar Chinese Spring [14,30,61] while ST99CH_1E4 (1E4) harbors AvrStb6 and is avirulent on Chinese Spring [42]. To visualize and distinguish pycnidia and hyphae of the two mixed strains, we used a 1E4 variant tagged with a codon-optimized version of cytoplasmic enhanced green fluorescent protein (eGFP) and a 3D7 variant tagged with the cytoplasmic monomeric Cherry (mCherry) [14,62–64]. Spores were incubated in 50 mL yeast sucrose broth (YSB, 10 g L^−1^ yeast extract, and 10 g L^−1^ sucrose supplemented with 50 mg mL^−1^ kanamycin) for 6 days at 18°C and 120 rpm. Spore cultures were filtered through two layers of sterile gauze, pelleted at 3273 g for 15 min, and resuspended into 15–25 mL of sterile water. The concentration of the spore inoculum was estimated using KOVA Glasstic counting chambers (Hycor Biomedical, Inc., California). Growth in axenic conditions of the strains used in infection experiments was analyzed by applying 3 μL drops of a dilution series 1: 10 starting from the sprayed concentration onto yeast malt sucrose agar (YMA, 4 g L^−1^ yeast extract, 4 g L^−1^ malt extract, 4 g L^−1^ sucrose and 12 g L^−1^ agar) media. Plates were assessed after 6 days of incubation at 18°C (S7 Fig).

### Whole plant spray infection assays

In all experiments, unless noted otherwise, seventeen-day-old wheat plants were spray-inoculated until run-off with either one strain (1E4, 3D7 or 1A5) or a combination of two strains (3D7+1E4 or 3D7+1A5) using 10 mL of the spore suspension containing 0.1% (v/v) Tween-20 (Sigma Aldrich) or a mock solution (water and 0.1% (v/v) Tween-20) per pot. In the sequential infection experiments plants were initially spray-inoculated with a mock solution or a spore suspension of 3D7-mCherry, and 7 days later the same plants were infected with 1E4-eGFP. As a positive control, 24-day-old plants were spray-inoculated with a spore suspension containing a mix of 1E4-eGFP and 3D7-mCherry. The concentration of the spore suspension was 10^6^ spores/mL for each strain, except for the confocal microscopy experiments in which 10^7^ spores/mL was used. After inoculation, pots were enclosed within a plastic bag for 72 h to ensure 100% relative humidity. 19–21 dpi (depending on the experiment) the second and third leaves were harvested and used for pycnidia quantification under the fluorescence stereomicroscope. Whole plant infection experiments shown in Fig 1A–1D were performed three times.

### Side-by-side infection and mechanical damage assays

In the side-by-side infection and mechanical-damage assays the second leaf was placed horizontally on a flat surface similar to what was previously described [65]. Elastic threads were used to hold the leaves on the flat surface and to segment the leaves into 7 sections of 2 cm length. In the side-by-side infection experiments each section was treated with either 1E4-eGFP or 3D7-mCherry. In control plants, mock solution was used instead of 3D7-mCherry, adjacent to 1E4-eGFP infected sections. In the mechanical damage assays leaves were pierced with a needle (Sterican Brown 0.45x12 mm BL/LB) every 1 cm along the second leaf or damaged by applying with a paintbrush a water solution containing celite (10 g/l) to a 7 cm section of the second leaf. Undamaged leaves were also included in the experiment. A spore suspension of 10^7^ spores/mL of each strain containing 0.1% (v/v) of Tween-20 or a mock solution were applied to the adaxial side of the leaves with a paintbrush, carefully avoiding contact with the elastic threads separating each leaf segment. Treated leaves were collected at 25 dpi for the side-by-side experiments and at 20 dpi for the mechanical damage experiments. In the side-by-side experiment, the tips (which were necrotic) were discarded from the analysis. Pycnidia of 1E4-eGFP were quantified by taking pictures with a fluorescence stereomicroscope. In the side-by-side experiment, we differentiated “pycnidia in proximity to 3D7” when in the same picture pycnidia from both strains were identified. “Pycnidia of 1E4 alone” were those that did not have any visible pycnidia of 3D7 in the proximity. These experiments were performed two times.

### Detached leaves infection assay

The top 2 cm of leaves of the 24-day-old plants were discarded and the adjacent 17 cm sections were placed flat onto water agar (1%) supplemented with 50 mg mL^−1^ kanamycin to keep the leaves moist. Spore suspensions of 1E4-eGFP, 3D7-mCherry, or a 1:1 mixture (10^6^ spores/mL for each strain) were applied with a paintbrush. Agar plates with the leaves were kept at the growing conditions described above for 12 days. At this stage, pycnidia were visible. Four images from random areas of each leaf were obtained with the fluorescence stereomicroscope and pycnidia of each strain were quantified manually.

### Visualization of oozing pycnidia and pycnidiospores

To visualize and quantify pycnidia, plants were analyzed between 19 and 25 dpi. Since mature pycnidia are heavily melanized, GFP emission can only be observed from oozing cirri. The first 2 cm from the leaf tip were discarded, and the adjacent 5 cm section was placed on YMA amended with 50 mg mL^−1^ kanamycin. The leaves were incubated at 18°C with 100% humidity for 24 hours to stimulate pycnidia oozing. A Leica M205 FCA stereomicroscope equipped with a Leica DFC 7000 T CCD color camera was used to capture images (Software Leica Application Suite X). The following filters were used to detect signal emissions: ET GFP (525 nm to 550 nm) and ET mCherry (630 nm to 675 nm). LED light without filters was used to obtain images on the bright field. The Fiji package from ImageJ (Version 2.3.0/1.53f, https://fiji.sc) was used to extract the pictures from the lif format and transform the images into jpg format. Pycnidiospores from single 1E4-eGFP pycnidia were collected with a sterile needle and dispersed into a droplet of sterile water placed on a microscope slide. A Leica DM2500 fluorescence microscope equipped with a Leica DFC3000 G gray-scale camera (Leica Microsystems, Wetzlar, Germany) and the filter blocks for GFP (480/40 nm excitation, 527/30 nm emission) and mCherry (580/20 nm excitation, 632/60 nm emission) were used to observe the pycnidiopores. The reproduction capacities (pycnidia quantification) of the virulent and the avirulent strains in single and in mixed infection were estimated.

### Confocal laser scanning microscopy

Confocal laser scanning microscopy was performed on an inverted Zeiss LSM 780 confocal microscope using two illumination sources, DPSS (561 nm) and Argon (488 nm) lasers. Signal detection for eGFP (494.95–535.07 nm); mCherry (625.61–643.42 nm) and chloroplast autofluorescence (656.01–681.98 nm) were set. Eleven days after infection, second leaves were collected, 3 cm of the tip of each leaf were discarded, and the adjacent section of 1.5–2 cm was mounted in 0.02% Tween-20. The entire adaxial side of the leaf segment was visually scanned under the microscope for penetration attempts. Images were processed using the Fiji package of ImageJ (Version 2.3.0/1.53f), and it included brightness and contrast adjustments, median filters (radius of 2 pixels), generation of maximum intensity z-projection, three-dimensional (3D) reconstruction, orthogonal projections, cropping, and addition of the scale bar. 3D reconstruction and orthogonal projections enabled us to localize the hyphae on the leaf section and to determine the stage of hyphal penetration (Fig 3A and 3B). In total at least 10 leaves per treatment and biological replicate were observed. In each biological replicate, independent inocula of each strain and pots were used. The experiment was repeated twice (Figs 2B and S3).

### RNA isolation and sequencing

Wheat plants were infected with 1E4-eGFP, 3D7-mCherry, the combination of both strains or with a mock solution. For each treatment, two leaves were pooled and a total of three biological replicates were obtained at 3 and 6 dpi. Three cm from the tip were discarded, and the adjacent 6 cm of the leaf were flash-frozen in liquid N_2_ and homogenized with zirconium beads (1.4 mm diameter) using the Bead-Rupture equipped with a cooling unit (Omni International, Kennesaw, GA, USA). RNA isolation was performed using GENEzol reagent (Geneaid Biotech, Taipei, Taiwan) following the manufacturer’s instructions. RNAeasy Mini Kit (Qiagen GmbH, Hilden, Germany) was used to purify the RNA, and DNA contamination was removed using the on-column DNase treatment of the RNase-Free DNase Set (Qiagen GmbH, Hilden, Germany). Ribosomal RNA was depleted by poly A enrichment, and DNA libraries were sequenced with Illumina NovaSeq 6000 using 150 bp paired-end reads. A total of 301.7 Gb of raw reads were produced. Adapters and low-quality reads were removed using Trimmomatic v0.35 [66] with the following parameters: ILLUMINACLIP:adapters.fa:2:28:10 LEADING:28 SLIDINGWINDOW:4:28 AVGQUAL:28 MINLEN:50. All raw sequence data generated in this study have been deposited in the NCBI Sequence Read Archive under accession number GSE232243.

### Transcriptomics profiling

Quality filtered reads were mapped onto the *Triticum aestivum* IWGSC transcriptome reference using Kallisto v0.46.1 with default parameters for paired-end reads [67]. Read counts were summarized using tximport v1.2.0 [68]. Differential gene expression and Gene Ontology (GO) enrichment analysis was performed with the R package DESeq2 [69] and topGO [70], respectively. Transcripts were considered to be differentially expressed compared to the controls (i.e., water sprayed plants) if DESeq2 p-value adjusted (padj) was < 0.05 and log2-fold change was > 0. Principal component analysis was performed with DESeq2 rlog-transformed normalized counts. GO annotations were retrieved from Ensembl using the R package BiomaRt [71]. We highlighted the GO annotations referring to defense responses into the category "defense-related genes" considering the following GO IDs: GO:0002215; GO:0002229; GO:0002679; GO:0006952; GO:0006968; GO:0031347; GO:0031348; GO:0031349; GO:0042742; GO:0050687; GO:0050688; GO:0050829; GO:0050832; GO:0051607; GO:0098542; GO:1900150; GO:1900367; GO:1900425; GO:1900426; GO:2000068; GO:2000071. Finally, we refined the GO annotation by extracting the protein sequences of the differentially expressed defense-related genes to search for sequence similarities in *Arabidopsis thaliana* using BLASTP [72].

### Statistical analysis

Statistical analyses were conducted in R [73]. The "mvnormtest" package from Shapiro-Wilk and the "car" package from Levene’s test were used to examine the normality of residuals and homogeneity of variance. In normally distributed data, a two-tailed student’s test (P < 0.05) was performed, except for the quantification of 1E4 successful penetration events in the substomatal cavity of Chinese Spring (Fig 2B), in which a one-tailed student’s test was performed. The statistical analysis for the quantification of 1E4 pycnidia per leaf (Fig 1C) was performed using a one-tailed Kruskal-Wallis test. Analysis of variance (ANOVA) followed by Tukey’s HSD post-hoc test was used to estimate differences in the reproduction of the avirulent strain 1E4 between single infection, sequential infection, and simultaneous infection (mixed infection). ANOVA followed by Tukey´s HSD post-hoc test was used to estimate significant differences between pycnidia quantification of the virulent strain 3D7 in single and in mixed infection on healthy plants. Counts of 1E4 pycnidia in the detached leaf experiment and in the sequential experiment were root square transformed to fulfill normality and homoscedasticity of residuals.

## Supporting information

S1 Fig *Zymoseptoria tritici* avirulent strain 1E4 asexual reproduction is higher in mixed infections with virulent strains. Barplots of the average number of 1E4 pycnidia per leaf (**A**) and per cm^2^ leaf (**C**) at 20 days post-infection (dpi) in single and mixed infections with 3D7. Three biological replicates (Replicate 2 is also included in Fig 1C). Infections were performed on the wheat cultivar Chinese Spring. Error bars represent the standard error of the mean. **B)** and **D)** The virulent strain 3D7 produces the same number of pycnidia regardless of the presence of the avirulent strain 1E4. Barplots show the average number of 3D7 pycnidia per leaf (B) or per cm^2^ of leaf (D) in each of the three biological replicates (Replicate 2 is also included in Fig 1D). Error bars represent the standard error of the mean. In A) and B) a total of 16 third leaves per replicate and treatment were analyzed.
(PDF)

S2 Fig Leaf damage enables pycnidia formation of the avirulent strain 1E4 of *Zymoseptoria tritici*. **A)** Pycnidia per wheat (cultivar Chinese Spring) leaf segment of the avirulent strain 1E4 in not damaged leaves and leaves poked or damaged with celite 30 min prior to infection. Error bars represent the standard error of the mean. In total, seven leaf segments of four second leaves per treatment were analyzed for each infection replicate. Results from two independent replicates are shown. Infections were evaluated 20 days post inoculation (dpi) with 1E4. Mixed infection on undamaged Chinese Spring leaves and mock-treated plants are included as a control. **B)** Representative pictures of leaves after the different treatments are shown.
(PDF)

S3 Fig *Zymoseptoria tritici* avirulent strain reaches the mesophyll in mixed infections with a virulent strain. Hyphae from the avirulent strain 1E4 reach wheat mesophyll cells more frequently in mixed infections with the virulent strain 3D7 (labeled with mCherry) than in single infections. The percentage of hyphae at each of the infection stages (I, II, III; Fig 2A) was estimated at 11 days after infection of Chinese Spring plants with 1E4-eGFP or with a mixture of 1E4-eGFP and 3D7-mCherry. Bars represent the average of three biological replicates, with standard errors. Asterisks indicate statistical differences according to two-tailed student’s test (P < 0.05). The results are from an independent repetition of the experiment shown in Fig 2B.
(PDF)

S4 Fig Rarefaction curve showing RNA sequencing reads mapped to *Zymoseptoria tritici* 1E4 (**A**) and 3D7 (**B**) transcriptomes at 3 and 6 dpi used as a proxy estimation for fungal biomass in single infection treatments.
(PDF)

S5 Fig Host responds specifically to *Zymoseptoria tritici* 1E4 single infection compared to 3D7 single and mixed infections. UpSet plot presenting the number of specific or shared differentially expressed genes up-regulated (A) or down-regulated (B) in wheat plants upon 1E4 single, 3D7 single and mixed infections at 3 and 6 dpi.
(PDF)

S6 Fig **Gene Ontology (GO) enrichment analysis of DEGs in wheat** at **A)** 3 dpi and **B)** 6 dpi, only significant GO enriched categories are shown, and "defense-related" GOs are highlighted in red. Numbers in circles represent GO IDs annotated, only GO categories containing at least 100 annotations in the wheat genome were displayed on the plot.
(PDF)

S7 Fig Fitness assay of the *Zymoseptoria tritici* strains used in the infection assays. Phenotypes of the strains used for the confocal microscopy infection assays in solid media. For each strain, 2 drops of 3 μL of fungal spore suspensions at a concentration of 5·10^6^, 5·10^5^, 5·10^4^ and 5·10^3^ spores mL^-1^ per strain (3D7 and 1E4) were inoculated on yeast-malt-sucrose agar (YMA) and incubated at 18°C for 5 days.
(PDF)

S1 Text Table A in S1 Text: Raw data of Figs 1C–1D and S1. Table B in S1 Text: Raw data of Fig 1E. Table C in S1 Text: Raw data of S2 Fig. Table D in S1 Text: Raw data of Fig 1H. Table E in S1 Text: Raw data of Fig 2. Table F in S1 Text: Raw data of S1 Fig. Table G in S1 Text: Differentially expressed genes in at least one condition in comparison to non-infected plants. Stb6 and Stb16q are highlighted in red and blue, respectively. Table H in S1 Text: Defense-related DEGs in 1E4 infection 3dpi or 6dpi, with functional annotations. Table I in S1 Text: Defense-related DEGs in 3D7 infection 3dpi or 6dpi, with functional annotations. Table J in S1 Text: Defense-related DEGs in mixed infection 3dpi or 6dpi, with functional annotations. Table K in S1 Text: Number of DEGs containing ’defense-related’ annotations from Fig 4H Table L in S1 Text: Biological process GO enrichment analysis of up-regulated genes at 3dpi. Table M in S1 Text: Biological process GO enrichment analysis of up-regulated genes at 6dpi. Table N in S1 Text: Similarity search for protein sequences of defense related DEGs in Arabidopsis using BLASTP.
(XLSX)

## References

[1] Tollenaere C, Susi H, Laine AL. Evolutionary and Epidemiological Implications of Multiple Infection in Plants. Trends Plant Sci. 2016;21: 80–90. doi: 10.1016/j.tplants.2015.10.014 26651920

[2] Tollenaere C, Lacombe S, Wonni I, Barro M, Ndougonna C, Gnacko F, et al. Virus-bacteria rice co-infection in africa: Field estimation, reciprocal effects, molecular mechanisms, and evolutionary implications. Front Plant Sci. 2017;8. doi: 10.3389/fpls.2017.00645 28507553 PMC5410622

[3] Abdullah AS, Moffat CS, Lopez-Ruiz FJ, Gibberd MR, Hamblin J, Zerihun A. Host–multi-pathogen warfare: Pathogen interactions in co-infected plants. Front Plant Sci. 2017;8: 1–12. doi: 10.3389/fpls.2017.01806 29118773 PMC5660990

[4] Rovenich H, Boshoven JC, Thomma BPHJ. Filamentous pathogen effector functions: Of pathogens, hosts and microbiomes. Curr Opin Plant Biol. 2014;20: 96–103. doi: 10.1016/j.pbi.2014.05.001 24879450

[5] Mideo N. Parasite adaptations to within-host competition. Trends Parasitol. 2009;25: 261–268. doi: 10.1016/j.pt.2009.03.001 19409846

[6] Holt RD, Bonsall MB. Apparent Competition. Annu Rev Ecol Evol Syst. 2017;48: 447–471. doi: 10.1146/annurev-ecolsys-110316-022628

[7] Alizon S, Van Baalen M. Multiple infections, immune dynamics, and the evolution of virulence. Am Nat. 2008;172. doi: 10.1086/590958 18702601

[8] Seybold H, Demetrowitsch TJ, Hassani MA, Szymczak S, Reim E, Haueisen J, et al. A fungal pathogen induces systemic susceptibility and systemic shifts in wheat metabolome and microbiome composition. Nat Commun. 2020;11: 1–12. doi: 10.1038/s41467-020-15633-x 32313046 PMC7171108

[9] Notz R, Maurhofer M, Dubach H, Haas D, Défago G. Fusaric acid-producing strains of *Fusarium oxysporum* alter 2,4-diacetylphloroglucinol biosynthetic gene expression in *Pseudomonas fluorescens* CHA0 in vitro and in the rhizosphere of wheat. Appl Environ Microbiol. 2002;68: 2229–2235. doi: 10.1128/AEM.68.5.2229–2235.2002 11976092 PMC127576

[10] Glazebrook J. Contrasting mechanisms of defense against biotrophic and necrotrophic pathogens. Annu Rev Phytopathol. 2005;43: 205–227. doi: 10.1146/annurev.phyto.43.040204.135923 16078883

[11] Gold A, Giraud T, Hood ME. Within-host competitive exclusion among species of the anther smut pathogen. BMC Ecol. 2009;9: 5–11. doi: 10.1186/1472-6785-9-11 19422703 PMC2688501

[12] Susi H, Barrès B, Vale PF, Laine AL. Co-infection alters population dynamics of infectious disease. Nat Commun. 2015;6. doi: 10.1038/ncomms6975 25569306 PMC4354079

[13] Laine AL. Context-dependent effects of induced resistance under co-infection in a plant-pathogen interaction. Evol Appl. 2011;4: 696–707. doi: 10.1111/j.1752-4571.2011.00194.x 25568016 PMC3352536

[14] Barrett LG, Zala M, Mikaberidze A, Alassimone J, Ahmad M, McDonald BA, et al. Mixed infections alter transmission potential in a fungal plant pathogen. Environ Microbiol. 2021;23: 2315–2330. doi: 10.1111/1462-2920.15417 33538383 PMC8248022

[15] Fiorin GL, Sanchéz-Vallet A, Thomazella DP de T, do Prado PFV, do Nascimento LC, Figueira AV de O, et al. Suppression of Plant Immunity by Fungal Chitinase-like Effectors. Curr Biol. 2018;28: 3023–3030.e5. doi: 10.1016/j.cub.2018.07.055 30220500

[16] He P, Chintamanani S, Chen Z, Zhu L, Kunkel BN, Alfano JR, et al. Activation of a COI1-dependent pathway in *Arabidopsis* by *Pseudomonas syringae* type III effectors and coronatine. Plant J. 2004;37: 589–602. doi: 10.1111/j.1365-313X.2003.01986.x 14756769

[17] Jiang S, Yao J, Ma KW, Zhou H, Song J, He SY, et al. Bacterial Effector Activates Jasmonate Signaling by Directly Targeting JAZ Transcriptional Repressors. PLoS Pathog. 2013;9. doi: 10.1371/journal.ppat.1003715 24204266 PMC3814404

[18] Lo Presti L, Lanver D, Schweizer G, Tanaka S, Liang L, Tollot M, et al. Fungal effectors and plant susceptibility. Annu Rev Plant Biol. 2015;66: 513–545. doi: 10.1146/annurev-arplant-043014-114623 25923844

[19] Haueisen J, Möller M, Eschenbrenner CJ, Grandaubert J, Seybold H, Adamiak H, et al. Highly flexible infection programs in a specialized wheat pathogen. Ecol Evol. 2019;9: 275–294. doi: 10.1002/ece3.4724 30680113 PMC6342133

[20] Haueisen J, Lorrain C, Stukenbrock EH. Molecular interactions of microbes and the plant phyllosphere. Cell Dialogues Holobiont. 2020; 267–285. doi: 10.1201/9780429277375-16

[21] Sánchez-Vallet A, Mesters JR, Thomma BPHJ. The battle for chitin recognition in plant-microbe interactions. FEMS Microbiol Rev. 2015;39: 171–183. doi: 10.1093/femsre/fuu003 25725011

[22] Gimenez-Ibanez S, Boter M, Fernández-Barbero G, Chini A, Rathjen JP, Solano R. The Bacterial Effector HopX1 Targets JAZ Transcriptional Repressors to Activate Jasmonate Signaling and Promote Infection in *Arabidopsis*. PLoS Biol. 2014;12. doi: 10.1371/journal.pbio.1001792 24558350 PMC3928049

[23] Martin F, Kamoun S. Effectors in Plant-Microbe Interactions. Effectors in Plant-Microbe Interactions. Wiley-Blackwell; 2012. doi: 10.1002/9781119949138

[24] Jones JDG, Dangl JL. The plant immune system. Nature. 2006;444: 323–329. doi: 10.1038/nature05286 17108957

[25] Kanyuka K, Rudd JJ. Cell surface immune receptors: the guardians of the plant’s extracellular spaces. Curr Opin Plant Biol. 2019;50: 1–8. doi: 10.1016/j.pbi.2019.02.005 30861483 PMC6731392

[26] Flor HH. Current Status of the Gene-For-Gene Concept. Annu Rev Phytopathol. 1971;9: 275–296. doi: 10.1146/annurev.py.09.090171.001423

[27] Bent AF, Mackey D. Elicitors, effectors, and R genes: The new paradigm and a lifetime supply of questions. Annu Rev Phytopathol. 2008;45: 399–436. doi: 10.1146/annurev.phyto.45.062806.094427 17506648

[28] McDowell JM, Dangl JL. Signal transduction in the plant immune response. Trends Biochem Sci. 2000;25: 79–82. doi: 10.1016/s0968-0004(99)01532-7 10664588

[29] Vlot AC, Sales JH, Lenk M, Bauer K, Brambilla A, Sommer A, et al. Systemic propagation of immunity in plants. New Phytol. 2021;229: 1234–1250. doi: 10.1111/nph.16953 32978988

[30] Linde CC, Zhan J, McDonald BA. Population structure of *Mycosphaerella graminicola*: From lesions to continents. Phytopathology. 2002;92: 946–955. doi: 10.1094/PHYTO.2002.92.9.946 18944019

[31] Zhan J, Linde CC, Jürgens T, Merz U, Steinebrunner F, McDonald BA. Variation for neutral markers is correlated with variation for quantitative traits in the plant pathogenic fungus *Mycosphaerella graminicola*. Mol Ecol. 2005;14: 2683–2693. doi: 10.1111/j.1365-294X.2005.02638.x 16029470

[32] McDonald BA, Suffert F, Bernasconi A, Mikaberidze A. How large and diverse are field populations of fungal plant pathogens? The case of *Zymoseptoria tritici*. Evol Appl. 2022. doi: 10.1111/EVA.13434 36187182 PMC9488677

[33] Sánchez-Vallet A, McDonald MC, Solomon PS, McDonald BA. Is *Zymoseptoria tritici*a hemibiotroph? Fungal Genet Biol. 2015;79: 29–32. doi: 10.1016/j.fgb.2015.04.001 26092787

[34] Kema GHJ, Yu DZ, Rijkenberg FHJ, Shaw MW, Baayen RP. Histology of the pathogenesis of *Mycosphaerella graminicola* in wheat. Phytopathology. 1996. pp. 777–786. doi: 10.1094/Phyto-86-777

[35] Duncan KE, Howard RJ. Cytological analysis of wheat infection by the leaf blotch pathogen *Mycosphaerella graminicola*. Mycol Res. 2000;104: 1074–1082. doi: 10.1017/S0953756299002294

[36] Brown JKM, Chartrain L, Lasserre-Zuber P, Saintenac C. Genetics of resistance to *Zymoseptoria tritici* and applications to wheat breeding. Fungal Genet Biol. 2015;79: 33–41. doi: 10.1016/j.fgb.2015.04.017 26092788 PMC4510316

[37] Saintenac C, Cambon F, Aouini L, Verstappen E, Ghaffary SMT, Poucet T, et al. A wheat cysteine-rich receptor-like kinase confers broad-spectrum resistance against Septoria tritici blotch. Nat Commun. 2021;12: 1–10. doi: 10.1038/s41467-020-20685-0 33469010 PMC7815785

[38] Yang N, McDonald MC, Solomon PS, Milgate AW. Genetic mapping of Stb19, a new resistance gene to *Zymoseptoria tritici* in wheat. Theor Appl Genet. 2018;131: 2765–2773. doi: 10.1007/s00122-018-3189-0 30238255

[39] Brading PA, Verstappen ECP, Kema GHJ, Brown JKM. A gene-for-gene relationship between wheat and *Mycosphaerella graminicola*, the Septoria tritici blotch pathogen. Phytopathology. 2002;92: 439–445. doi: 10.1094/PHYTO.2002.92.4.439 18942957

[40] Saintenac C, Lee WS, Cambon F, Rudd JJ, King RC, Marande W, et al. Wheat receptor-kinase-like protein Stb6 controls gene-for-gene resistance to fungal pathogen *Zymoseptoria tritici*. Nat Genet. 2018;50: 368–374. doi: 10.1038/s41588-018-0051-x 29434355

[41] Kema GHJ, Gohari AM, Aouini L, Gibriel HAY, Ware SB, Bosch F Van Den, et al. Stress and sexual reproduction affect the dynamics of the wheat pathogen effector AvrStb6 and strobilurin resistance. Nat Genet. 2018;50. doi: 10.1038/s41588-018-0052-9 29434356

[42] Zhong Z, Marcel TC, Hartmann FE, Ma X, Plissonneau C, Zala M, et al. A small secreted protein in *Zymoseptoria tritici* is responsible for avirulence on wheat cultivars carrying the Stb6 resistance gene. New Phytol. 2017;214: 619–631. doi: 10.1111/nph.14434 28164301

[43] Battache M, Lebrun MH, Sakai K, Soudière O, Cambon F, Langin T, et al. Blocked at the Stomatal Gate, a Key Step of Wheat Stb16q-Mediated Resistance to *Zymoseptoria tritici*. Front Plant Sci. 2022;13: 1–14. doi: 10.3389/fpls.2022.921074 35832231 PMC9271956

[44] Ghiasi Noei F, Imami M, Didaran F, Ghanbari MA, Zamani E, Ebrahimi A, et al. Stb6 mediates stomatal immunity, photosynthetic functionality, and the antioxidant system during the *Zymoseptoria tritici*-wheat interaction. Front Plant Sci. 2022;13: 1–16. doi: 10.3389/fpls.2022.1004691 36388590 PMC9645118

[45] Brunner PC, McDonald BA. Evolutionary analyses of the avirulence effector AvrStb6 in global populations of *Zymoseptoria tritici* identify candidate amino acids involved in recognition. Mol Plant Pathol. 2018;19: 1836–1846. doi: 10.1111/mpp.12662 29363872 PMC6637991

[46] Orellana-Torrejon C, Vidal T, Gazeau G, Boixel AL, Gélisse S, Lageyre J, et al. Multiple scenarios for sexual crosses in the fungal pathogen *Zymoseptoria tritici* on wheat residues: Potential consequences for virulence gene transmission. Fungal Genet Biol. 2022;163. doi: 10.1016/j.fgb.2022.103744 36209959

[47] Liu G, Kennedy R, Greenshields DL, Peng G, Forseille L, Selvaraj G, et al. Detached and Attached *Arabidopsis* Leaf Assays Reveal Distinctive Defense Responses Against Hemibiotrophic *Colletotrichum* spp. Mol Plant-Microbe Interact. 2007;20: 1308–1319. doi: 10.1094/MPMI-20-10-1308 17918632

[48] Meile L, Garrido-Arandia M, Bernasconi Z, Peter J, Schneller A, Bernasconi A, et al. Natural variation in Avr3D1 from *Zymoseptoria* sp. contributes to quantitative gene-for-gene resistance and to host specificity. New Phytol. 2023;238: 1562–1577. doi: 10.1111/nph.18690 36529883

[49] Cook DE, Mesarich CH, Thomma BPHJ. Understanding Plant Immunity as a Surveillance System to Detect Invasion. Annu Rev Phytopathol. 2015;53: 541–563. doi: 10.1146/annurev-phyto-080614-120114 26047564

[50] Komar V, Vigne E, Demangeat G, Lemaire O, Fuchs M. Cross-protection as control strategy against Grapevine fanleaf virus in naturally infected vineyards. Plant Dis. 2008;92: 1689–1694. doi: 10.1094/PDIS-92-12-1689 30764294

[51] Fones HN, Eyles CJ, Kay W, Cowper J, Gurr SJ. A role for random, humidity-dependent epiphytic growth prior to invasion of wheat by *Zymoseptoria tritici*. Fungal Genet Biol. 2017;106: 51–60. doi: 10.1016/j.fgb.2017.07.002 28694096 PMC5556705

[52] Fantozzi E, Kilaru S, Gurr SJ, Steinberg G. Asynchronous development of *Zymoseptoria tritici* infection in wheat. Fungal Genet Biol. 2021;146: 103504. doi: 10.1016/j.fgb.2020.103504 33326850 PMC7812371

[53] Fones HN, Soanes D, Gurr SJ. Epiphytic proliferation of *Zymoseptoria tritici* isolates on resistant wheat leaves. Fungal Genet Biol. 2023;168: 103822. doi: 10.1016/j.fgb.2023.103822 37343618

[54] Plett JM, Martin FM. Know your enemy, embrace your friend: using omics to understand how plants respond differently to pathogenic and mutualistic microorganisms. Plant J. 2018;93: 729–746. doi: 10.1111/tpj.13802 29265527

[55] Suffert F, Delestre G, Carpentier F, Gazeau G, Walker AS, Gélisse S, et al. Fashionably late partners have more fruitful encounters: Impact of the timing of co-infection and pathogenicity on sexual reproduction in *Zymoseptoria tritici*. Fungal Genet Biol. 2016;92: 40–49. doi: 10.1016/j.fgb.2016.05.004 27178650

[56] Kerdraon L, Barret M, Laval V, Suffert F. Differential dynamics of microbial community networks help identify microorganisms interacting with residue-borne pathogens: The case of *Zymoseptoria tritici* in wheat. Microbiome. 2019;7: 1–17. doi: 10.1186/s40168-019-0736-0 31470910 PMC6717385

[57] Weiberg A, Wang M, Lin FM, Zhao H, Zhang Z, Kaloshian I, et al. Fungal small RNAs suppress plant immunity by hijacking host RNA interference pathways. Science (80-). 2013;342: 118–123. doi: 10.1126/science.1239705 24092744 PMC4096153

[58] Brown JKM, Tellier A. Plant-parasite coevolution: Bridging the gap between genetics and ecology. Annu Rev Phytopathol. 2011;49: 345–367. doi: 10.1146/annurev-phyto-072910-095301 21513455

[59] Thrall PH, Barrett LG, Dodds PN, Burdon JJ. Epidemiological and evolutionary outcomes in gene-for-gene and matching allele models. Front Plant Sci. 2016;6: 1–12. doi: 10.3389/fpls.2015.01084 26779200 PMC4703789

[60] Stephens C, Ölmez F, Blyth H, McDonald M, Bansal A, Turgay EB, et al. Remarkable recent changes in the genetic diversity of the avirulence gene AvrStb6 in global populations of the wheat pathogen *Zymoseptoria tritici*. Mol Plant Pathol. 2021;22: 1121–1133. doi: 10.1111/mpp.13101 34258838 PMC8358995

[61] Croll D, Zala M, McDonald BA. Breakage-fusion-bridge Cycles and Large Insertions Contribute to the Rapid Evolution of Accessory Chromosomes in a Fungal Pathogen. PLoS Genet. 2013;9. doi: 10.1371/journal.pgen.1003567 23785303 PMC3681731

[62] Meile L, Croll D, Brunner PC, Plissonneau C, Hartmann FE, McDonald BA, et al. A fungal avirulence factor encoded in a highly plastic genomic region triggers partial resistance to septoria tritici blotch. New Phytol. 2018;219: 1048–1061. doi: 10.1111/nph.15180 29693722 PMC6055703

[63] Kilaru S, Steinberg G. Yeast recombination-based cloning as an efficient way of constructing vectors for *Zymoseptoria tritici*. Fungal Genet Biol. 2015;79: 76–83. doi: 10.1016/j.fgb.2015.03.017 26092792 PMC4502459

[64] Schuster M, Kilaru S, Guo M, Sommerauer M, Lin C, Steinberg G. Red fluorescent proteins for imaging *Zymoseptoria tritici* during invasion of wheat. Fungal Genet Biol. 2015;79: 132–140. doi: 10.1016/j.fgb.2015.03.025 26092800 PMC4502450

[65] Karisto P, Dora S, Mikaberidze A. Measurement of infection efficiency of a major wheat pathogen using time-resolved imaging of disease progress. Plant Pathol. 2019;68: 163–172. doi: 10.1111/ppa.12932

[66] Bolger AM, Lohse M, Usadel B. Trimmomatic: A flexible trimmer for Illumina sequence data. Bioinformatics. 2014;30: 2114–2120. doi: 10.1093/bioinformatics/btu170 24695404 PMC4103590

[67] Bray NL, Pimentel H, Melsted P, Pachter L. Near-optimal probabilistic RNA-seq quantification. Nat Biotechnol. 2016;34: 525–527. doi: 10.1038/nbt.3519 27043002

[68] Soneson C, Love MI, Robinson MD. Differential analyses for RNA-seq: Transcript-level estimates improve gene-level inferences. F1000Research. 2016;4: 1–19. doi: 10.12688/F1000RESEARCH.7563.2 26925227 PMC4712774

[69] Love MI, Huber W, Anders S. Moderated estimation of fold change and dispersion for RNA-seq data with DESeq2. Genome Biol. 2014;15: 1–21. doi: 10.1186/s13059-014-0550-8 25516281 PMC4302049

[70] Adrian Alexa JR. topGO: Enrichment Analysis for Gene Ontology. 2021. doi: 10.18129/B9.bioc.topGO

[71] Durinck S, Spellman PT, Birney E, Huber W. Mapping identifiers for the integration of genomic datasets with the R/ Bioconductor package biomaRt. Nat Protoc. 2009;4: 1184–1191. doi: 10.1038/nprot.2009.97 19617889 PMC3159387

[72] Sayers EW, Bolton EE, Brister JR, Canese K, Chan J, Comeau DC, et al. Database resources of the national center for biotechnology information. Nucleic Acids Res. 2022;50: D20–D26. doi: 10.1093/nar/gkab1112 34850941 PMC8728269

[73] R Core Team (2022). R: A language and environment for statistical computing. R Foundation for Statistical Computing, Vienna, Austria. https://www.R-project.org/.

