# Supplementary figures and images for: Virulent strains of Zymoseptoria tritici suppress the host immune response and facilitate the success of avirulent strains in mixed infections
Source: PLoS Pathog. 2023 Nov 16;19(11):e1011767. doi: 10.1371/journal.ppat.1011767 (PMC10721197; doi:10.1371/journal.ppat.1011767)

S1 Fig

A

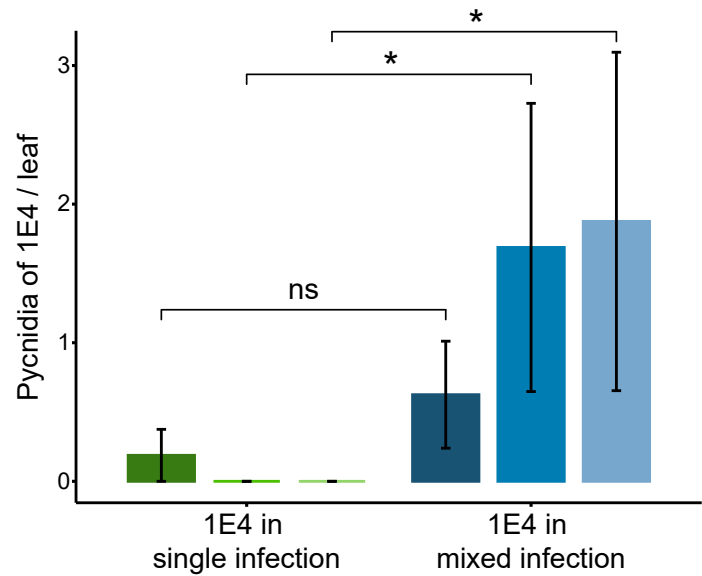

B

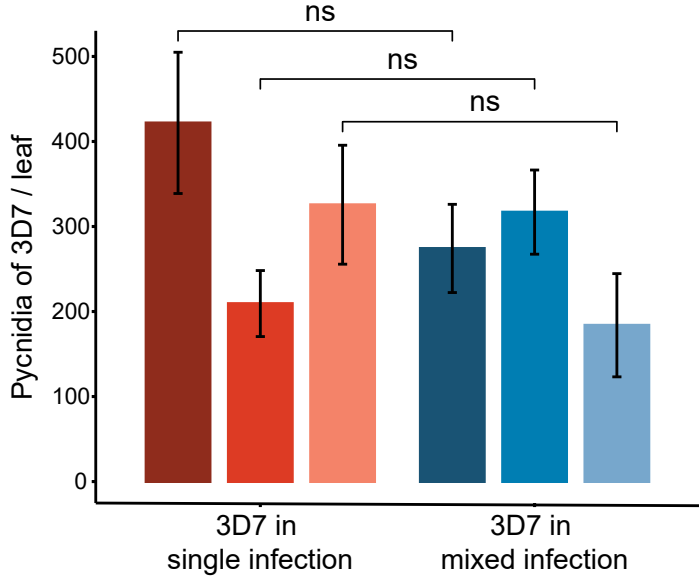

C

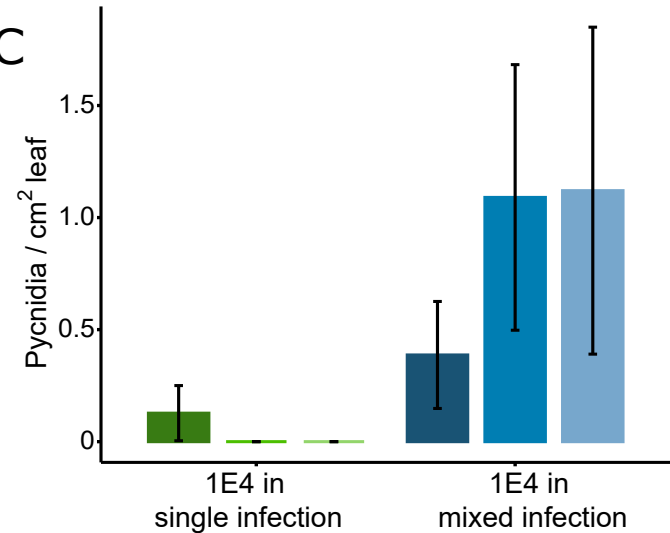

D

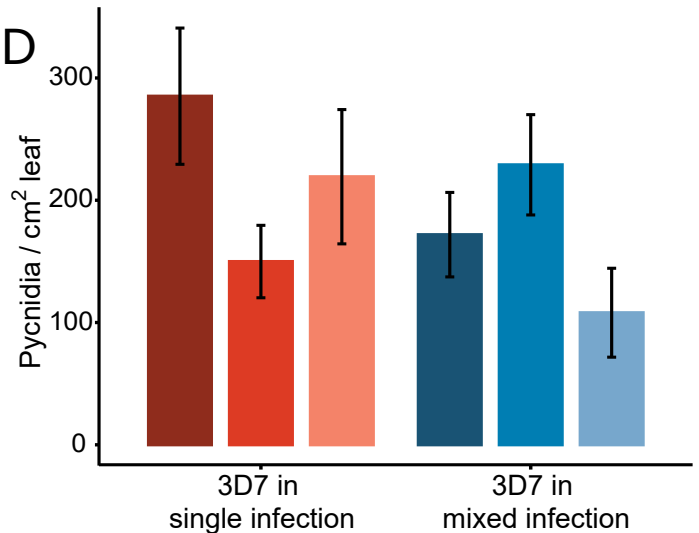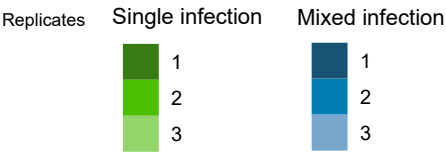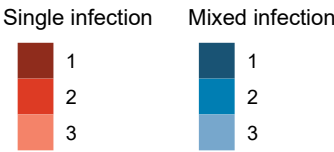

Supplement: S1 Fig — Barplots of the average number of 1E4 pycnidia per leaf (A) and per cm2 leaf (C) at 20 days post-infection (dpi) in single and mixed infections with 3D7. Three biological replicates (Replicate 2 is also included in Fig 1C). Infections were performed on the wheat cultivar Chinese Spring. Error bars represent the standard error of the mean. B) and D) The virulent strain 3D7 produces the same number of pycnidia regardless of the presence of the avirulent strain 1E4. Barplots show the average number of 3D7 pycnidia per leaf (B) or per cm2 of leaf (D) in each of the three biological replicates (Replicate 2 is also included in Fig 1D). Error bars represent the standard error of the mean. In A) and B) a total of 16 third leaves per replicate and treatment were analyzed. (PDF) [file ppat.1011767.s001.pdf]

# S2 Fig

A

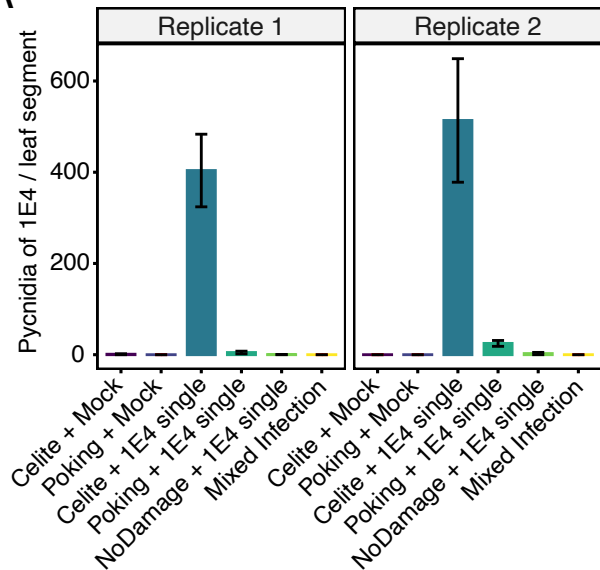

B

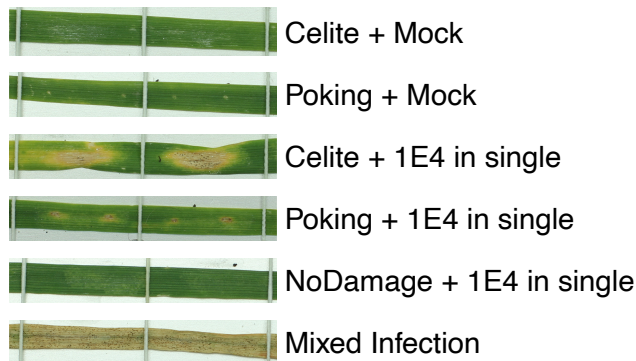

Supplement: S2 Fig — A) Pycnidia per wheat (cultivar Chinese Spring) leaf segment of the avirulent strain 1E4 in not damaged leaves and leaves poked or damaged with celite 30 min prior to infection. Error bars represent the standard error of the mean. In total, seven leaf segments of four second leaves per treatment were analyzed for each infection replicate. Results from two independent replicates are shown. Infections were evaluated 20 days post inoculation (dpi) with 1E4. Mixed infection on undamaged Chinese Spring leaves and mock-treated plants are included as a control. B) Representative pictures of leaves after the different treatments are shown. (PDF) [file ppat.1011767.s002.pdf]

Second Confocal session

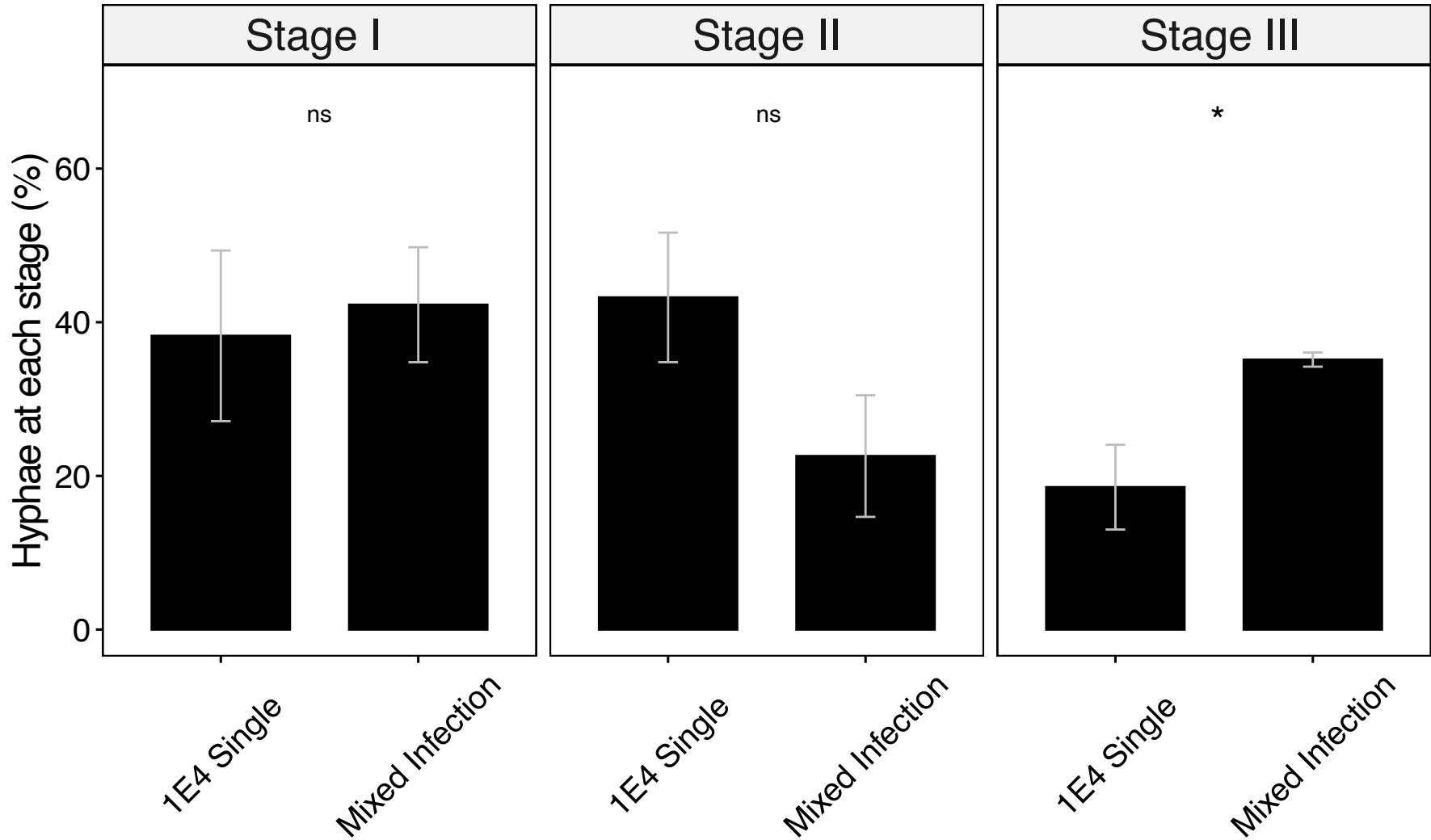

Supplement: S3 Fig — Hyphae from the avirulent strain 1E4 reach wheat mesophyll cells more frequently in mixed infections with the virulent strain 3D7 (labeled with mCherry) than in single infections. The percentage of hyphae at each of the infection stages (I, II, III; Fig 2A) was estimated at 11 days after infection of Chinese Spring plants with 1E4-eGFP or with a mixture of 1E4-eGFP and 3D7-mCherry. Bars represent the average of three biological replicates, with standard errors. Asterisks indicate statistical differences according to two-tailed student’s test (P < 0.05). The results are from an independent repetition of the experiment shown in Fig 2B. (PDF) [file ppat.1011767.s003.pdf]

# S4 Fig

A

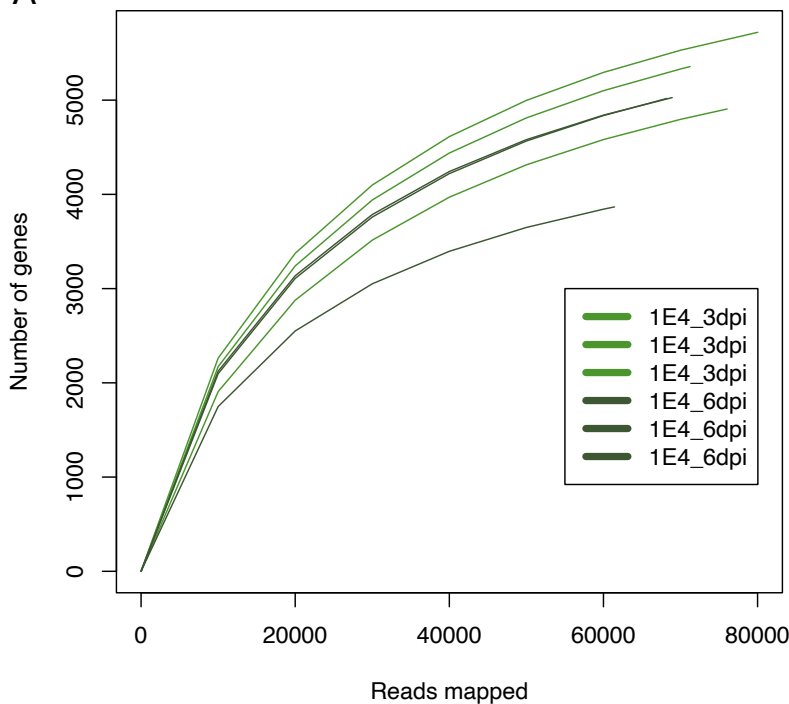

B

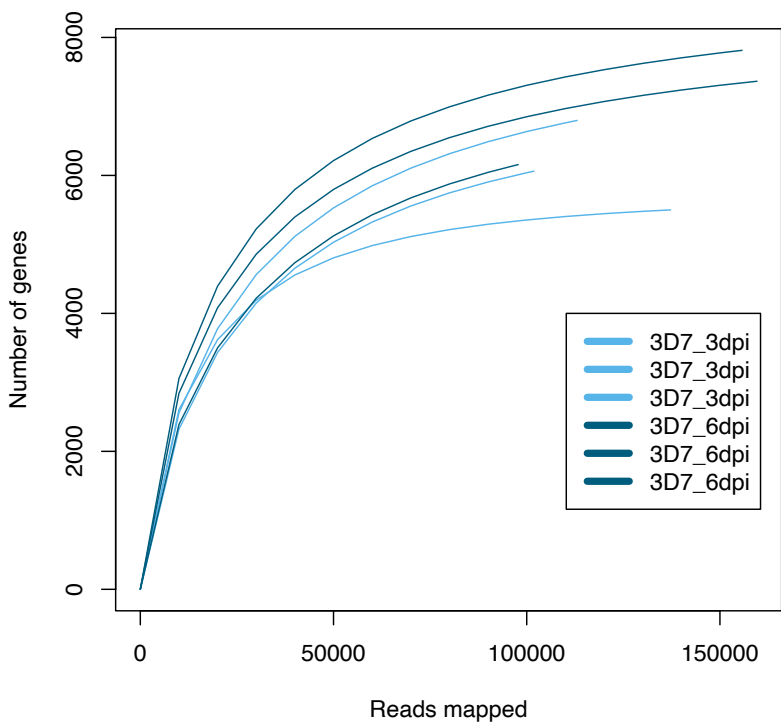

Supplement: S4 Fig — 1E4 (A) and 3D7 (B) transcriptomes at 3 and 6 dpi used as a proxy estimation for fungal biomass in single infection treatments. (PDF) [file ppat.1011767.s004.pdf]

A

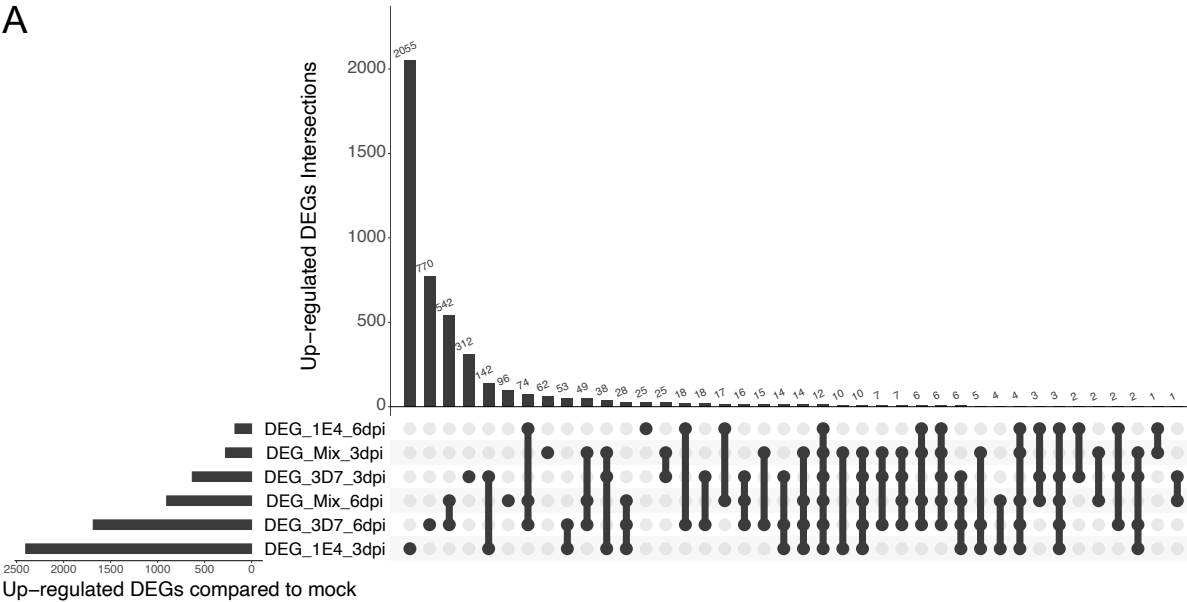

B

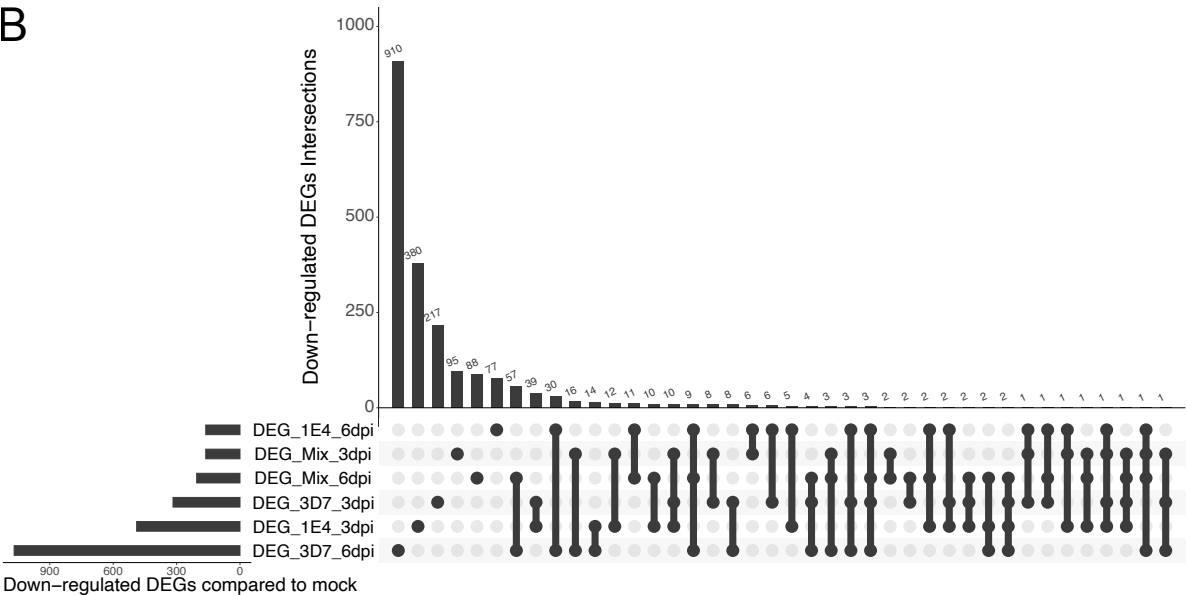

Supplement: S5 Fig — UpSet plot presenting the number of specific or shared differentially expressed genes up-regulated (A) or down-regulated (B) in wheat plants upon 1E4 single, 3D7 single and mixed infections at 3 and 6 dpi. (PDF) [file ppat.1011767.s005.pdf]

Figure S6

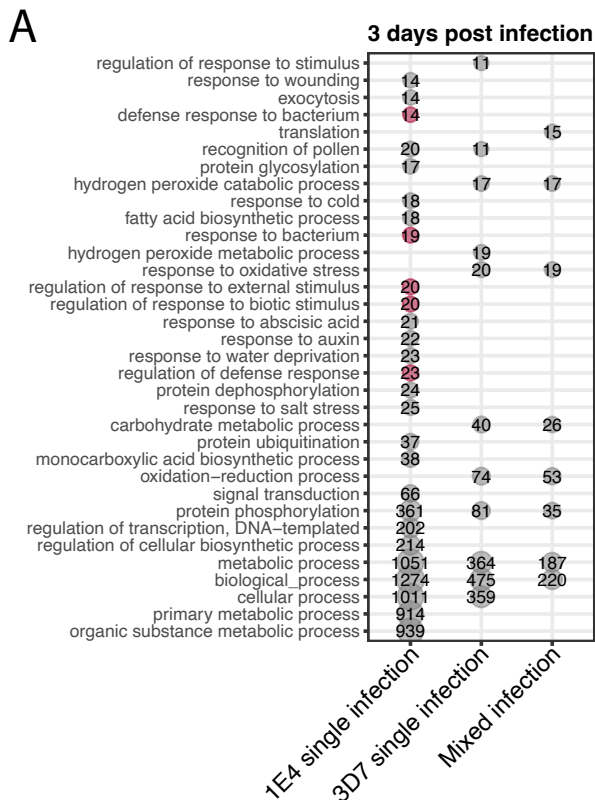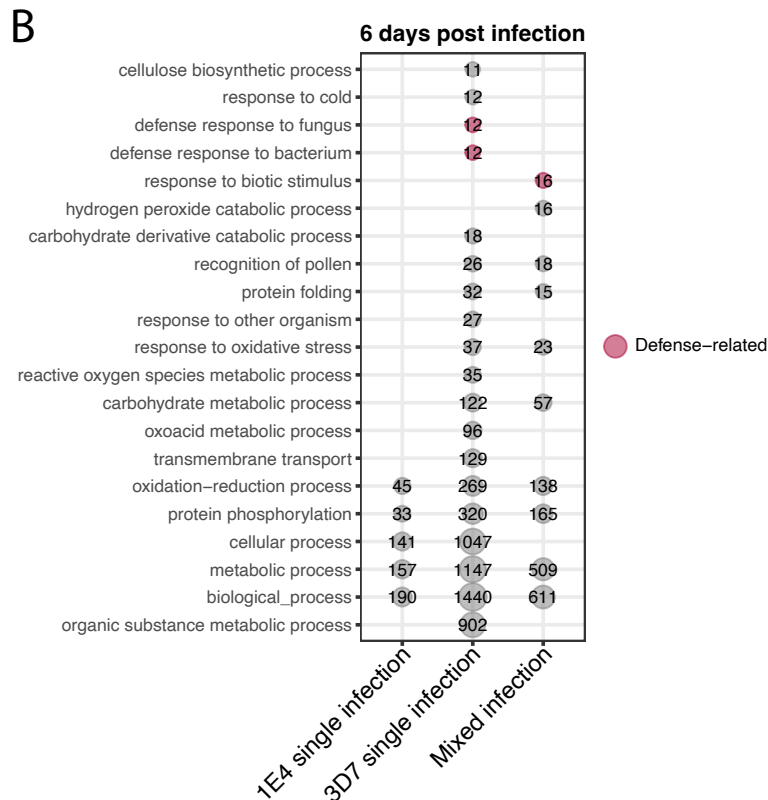

Supplement: S6 Fig — Gene Ontology (GO) enrichment analysis of DEGs in wheat at A) 3 dpi and B) 6 dpi, only significant GO enriched categories are shown, and "defense-related" GOs are highlighted in red. Numbers in circles represent GO IDs annotated, only GO categories containing at least 100 annotations in the wheat genome were displayed on the plot. (PDF) [file ppat.1011767.s006.pdf]

S7 Fig

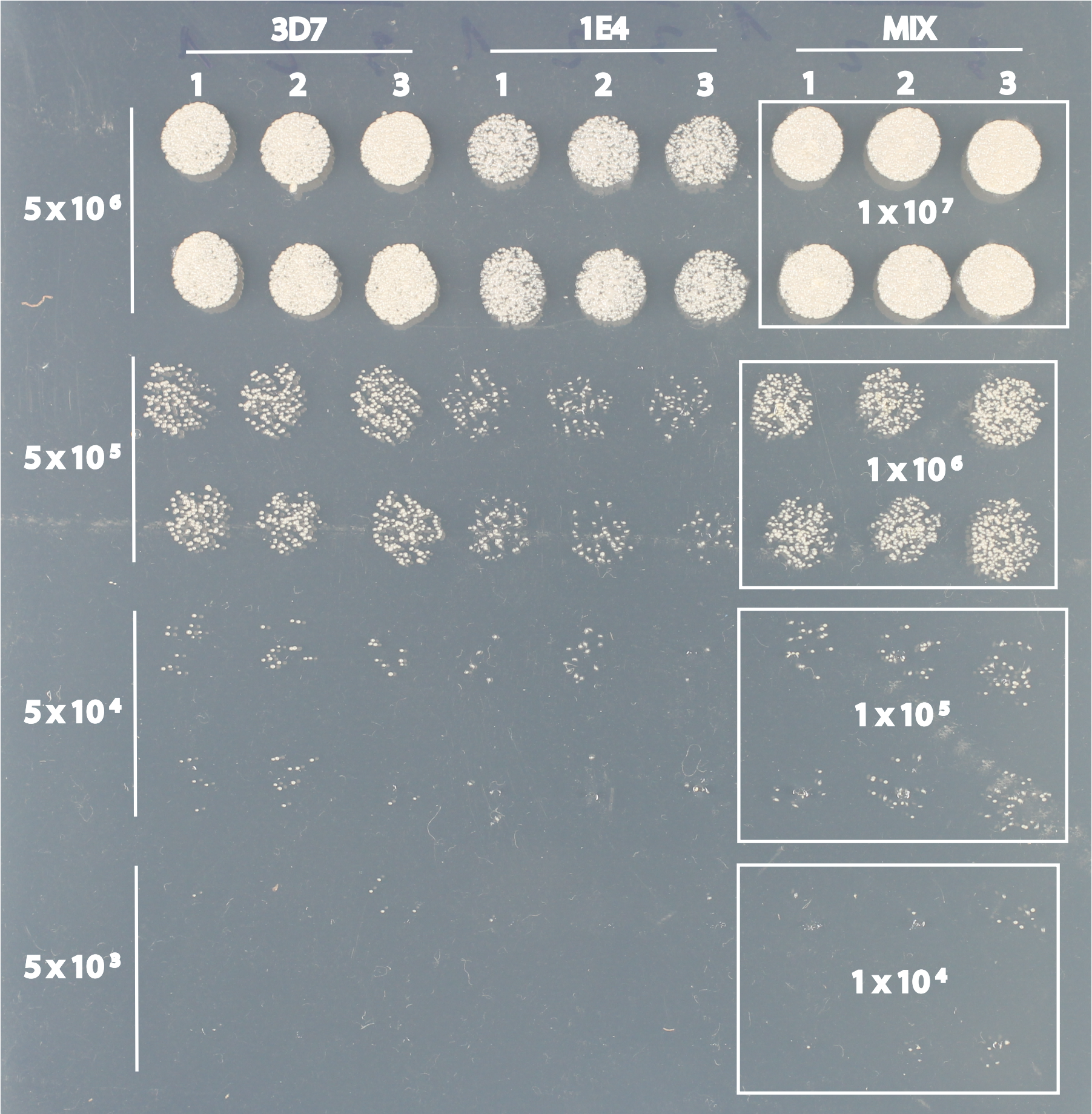

Supplement: S7 Fig — Phenotypes of the strains used for the confocal microscopy infection assays in solid media. For each strain, 2 drops of 3 μL of fungal spore suspensions at a concentration of 5·106, 5·105, 5·104 and 5·103 spores mL-1 per strain (3D7 and 1E4) were inoculated on yeast-malt-sucrose agar (YMA) and incubated at 18°C for 5 days. (PDF) [file ppat.1011767.s007.pdf]
